# Functional PDMS Elastomers: Bulk Composites, Surface Engineering, and Precision Fabrication

**DOI:** 10.1002/advs.202304506

**Published:** 2023-10-09

**Authors:** Shaopeng Li, Jiaqi Zhang, Jian He, Weiping Liu, YuHuang Wang, Zhongjie Huang, Huan Pang, Yiwang Chen

**Affiliations:** ^1^ State Key Laboratory for Modification of Chemical Fibers and Polymer Materials College of Materials Science and Engineering Donghua University Shanghai 201620 China; ^2^ Yizhi Technology (Shanghai) Co., Ltd No. 99 Danba Road, Putuo District Shanghai 200062 China; ^3^ Center for Composites COMAC Shanghai Aircraft Manufacturing Co. Ltd Shanghai 201620 China; ^4^ Department of Chemistry and Biochemistry University of Maryland College Park MD 20742 USA; ^5^ Maryland NanoCenter University of Maryland College Park MD 20742 USA; ^6^ School of Chemistry and Chemical Engineering Yangzhou University Yangzhou Jiangsu 225009 China; ^7^ National Engineering Research Center for Carbohydrate Synthesis/Key Lab of Fluorine and Silicon for Energy Materials and Chemistry of Ministry of Education Jiangxi Normal University 99 Ziyang Avenue Nanchang 330022 China

**Keywords:** additive manufacturing, flexible electronics, polydimethylsiloxane (PDMS), programmable materials, sensors, silicone, soft actuators

## Abstract

Polydimethylsiloxane (PDMS)—the simplest and most common silicone compound—exemplifies the central characteristics of its class and has attracted tremendous research attention. The development of PDMS‐based materials is a vivid reflection of the modern industry. In recent years, PDMS has stood out as the material of choice for various emerging technologies. The rapid improvement in bulk modification strategies and multifunctional surfaces has enabled a whole new generation of PDMS‐based materials and devices, facilitating, and even transforming enormous applications, including flexible electronics, superwetting surfaces, soft actuators, wearable and implantable sensors, biomedicals, and autonomous robotics. This paper reviews the latest advances in the field of PDMS‐based functional materials, with a focus on the added functionality and their use as programmable materials for smart devices. Recent breakthroughs regarding instant crosslinking and additive manufacturing are featured, and exciting opportunities for future research are highlighted. This review provides a quick entrance to this rapidly evolving field and will help guide the rational design of next‐generation soft materials and devices.

## Graphical abstract

Polydimethylsiloxane (PDMS), transforms from the central component in children's toy—silly putty, to the material of choice for many emerging technologies such as flexible electronics, soft actuators, and more, thanks to the advancement in composite science, multifunctional surfaces, and additive manufacturing. This article reviews latest progress in PDMS‐based elastomers and the integrated functionalities, highlighting the opportunities as well as challenges.

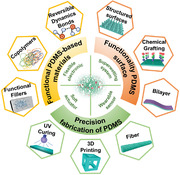

## 1 Introduction

**Figure 1:**
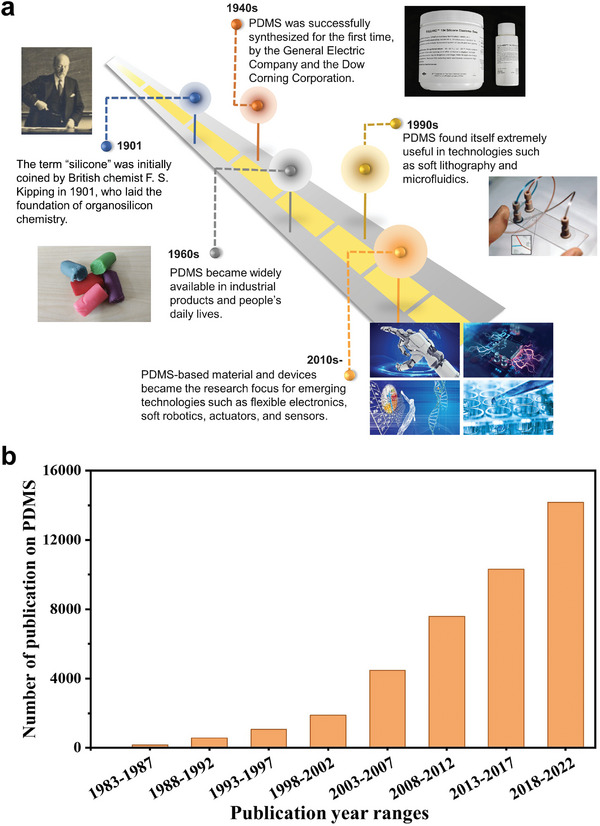
Evolution of PDMS‐based elastomer. a) Frederic S. Kipping, the founder of organosilicon chemistry, introduced the term “silicone” in 1901. He successfully synthesized hydrolyzable silanes with various functionalities using the Grignard reaction. Image 1901: Reproduced with permission.^[^ ^365^ ^]^ Copyright 2010, Springer. PDMS was first successfully synthesized in the 1940s owing to the seminal efforts of General Electric and Dow Corning, and by the 1960s, it was widely available in industrial products and consumer goods. In the 1990s, PDMS became the material of choice for new technologies such as soft lithography and microfluidics. Images 1940, 1960 and 1990: Reproduced with permission.^[^ ^262^ ^]^ Copyright 2017, American Chemical Society. By the 21st century, especially during the past decade, interest in PDMS has soared and this compound has been further modified for use in emerging technologies, including flexible electronics, bioengineering, actuators, and structured surfaces. Image 2010: Reproduced with permission. Photo courtesy of 51yuansu.com. b) Research trends in the field of PDMS are illustrated by the number of publications per year (Data from the Web of Science).

Silicone, also known as polysiloxane, is a synthetic polymer with backbone chains of alternating Si and O atoms. Silicone is a chemically inert, thermally stable substance with excellent water and oxidation resistance. It is used in a wide range of products, such as everyday chemicals, sealants, and biomedical implants. The simplest and most common silicone compound, polydimethylsiloxane (PDMS), represents the central characteristics of this class of materials and has been extensively researched. Based on the degree of interlinking, PDMS exists as a colorless, viscous fluid (i.e., lubricants) or as an elastomer film. The advancement of PDMS‐based materials has experienced several stages, as shown in **Figure** **1**a, and the advancements vividly reflect modern industries' development. The term “silicone” was initially coined by British chemist Frederic S. Kipping in 1901^[^ ^1^ ^]^ because he thought the Si and O atoms were connected with a double bond similar to a ketone. (In fact, the 3p electrons of Si do not overlap effectively with the 2p electrons of C or O, so the Si═O or Si═C multiple bonds are not commonly found in stable molecules.) In the 1940s, PDMS was successfully synthesized for the first time owing to the pioneering efforts of the General Electric Company and the Dow Corning Corporation. However, until the 1960s, PDMS was not widely available in industrial products and people's daily lives (such as in silly putty). In the 1990s, PDMS applications expanded to newer technologies such as soft lithography^[^ ^2^, ^3^, ^4^ ^]^ and microfluidics.^[^ ^5^ ^]^ In the 21st century, especially in the past decade, PDMS has been considered the material of choice for many emerging technologies, including flexible electronics, biomedical devices, actuators, and structured surfaces.^[^ ^6^, ^7^, ^8^, ^9^, ^10^, ^11^, ^12^ ^]^

PDMS is well‐known for its extraordinary optical transparency, biocompatibility, processability, and thermal and chemical stability. However, along with the rapid development of science and technology in modern society, conventional silicones' mechanical and physicochemical properties cannot fully meet the needs for diverse implementation scenarios of advanced technological applications. A new generation of silicone elastomers with various functionalities and actuating/sensing capabilities is required. Therefore, significant advancements in functional silicones have been made in the past several years. The modification of PDMS generally includes the innovative design of novel side chains or crosslinkers, the incorporation of functional fillers (with electrical, optical, thermal, magnetic, or mechanical properties), and the fabrication of advanced micro‐ and nanostructures. Through these improvements, the elastomer composites' mechanical, optical, electrical, thermal, chemical, and surface properties can be systematically manipulated and tailored across a wide range and precisely controlled for specific needs or novel applications.

As shown in Figure 1b, the research interest in PDMS‐related materials and systems has exploded in the past few years, coinciding with the rapid development of soft functional materials and flexible electronics. Therefore, a critical review of this rapidly evolving field is urgently needed, especially on the added functionalities, use as smart materials in multidisciplinary fields, and cutting‐edge additive manufacturing paradigms. We notice that there exist a handful of reviews on PDMS‐related materials, either focused on a specific application (e.g., dielectric transducer,^[^ ^13^ ^]^ protective coating,^[^ ^14^ ^]^ microfluidic devices,^[^ ^15^ ^]^ substrates for stretchable electronics^[^ ^16^ ^]^), or regarding the earlier‐stage development in this field.^[^ ^17^, ^18^ ^]^ In this review, we aim to condense many concepts from various communities that use PDMS into one place, and provide critical insights on the latest (e.g., in the past three years) advances in this field. Additive manufacturing represents a revolutionary strategy for the three‐dimensional (3D) production of materials with digital design and high energy efficiency and is gaining increasing momentum^[^ ^19^ ^]^ and 3D printing of PDMS, despite a number of inherent challenges associated with the material, already has been demonstrated to be feasible. In this comprehensive review, we aim to elucidate the versatile aspects of the PDMS‐based elastomer as a material uniquely positioned for a plethora of fields. In Section 2, we present an introduction to the fundamental properties that underpin PDMS's growing prominence in the realm of multifaceted applications. In Section 3, we delve into the development of PDMS‐based functional materials. On one hand, the composites and the modification and compounding protocols will be discussed, categorized by the integrated functionalities (such as electrical, optical, thermal, magnetic, mechanical, and chemical properties). On the other hand, we will explore the functionality of the PDMS surface, with an emphasis on the superwetting features and bimorph actuators that have garnered significant attention in recent years. In Section 4, we highlight the state‐of‐the‐art breakthroughs in expediting crosslinking and additive manufacturing processes of PDMS‐based materials, which hold immense potential in a variety of applications. Lastly, we provide a concise summary of our findings, pinpointing the most promising research directions and uncharted territories ripe for future investigation. We hope that this timely, in‐depth review serves both as a fundamental starting point and a handbook for researchers interested in understanding this rapidly evolving field.

## 2 Fundamental Properties of PDMS

### 2.1 Cross‐Linking Chemistry

**Figure 2:**
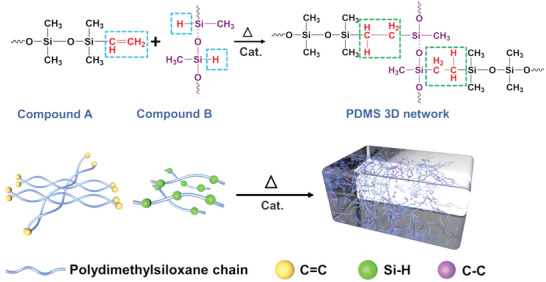
Scheme showing the most widely employed crosslinking mechanism of PDMS. The polymerization is based on a platinum‐catalyzed process between a dimethylsiloxane oligomer with vinyl terminal groups and a curing agent (dimethyl methylhydrogen siloxane) containing a Si–H bond. The crosslinking can be accelerated by heat.

PDMS can be prepared based on various chemistries, such as hydrosilylation addition, condensation reaction, and ultraviolet (UV) curing.^[^ ^20^, ^21^ ^]^ Among these methods, thermal crosslinking through hydrosilylation addition between vinyl terminals (CH_2_═CH–) and silicon hydride (Si–H) is most widely employed (such as in Dow Corning Sylgard 184 series) and remains an optimal option for many applications owing to its simple, non‐toxic, and byproduct‐free curing process, which allows for facile preparation with high molding accuracy.^[^ ^22^ ^]^ The vinyl functional (Sylgard 184‐like) PDMS usually consists of two parts (**Figure** **2**), and the polymerization is based on a platinum‐catalyzed process between a dimethylsiloxane oligomer with vinyl terminal groups and a curing agent (dimethyl methylhydrogen siloxane) containing Si–H bond. Crosslinking can be accelerated at elevated temperatures. It should be noted that multiple categories of species can inhibit hydrosilylation crosslinking.^[^ ^22^ ^]^ Lone pairs of specific nitrogen or sulfur compounds tend to complex with and defunctionalize the Pt catalyst,^[^ ^23^, ^24^ ^]^ and the Si–H bond can be added onto multiple unsaturated bonds if organic molecules containing alkenes, alkynes, imines, or carbonyls are present.^[^ ^25^, ^26^ ^]^

### 2.2 Physical and Chemical Properties

The general physical properties of PDMS are listed in **Table** **1**. PDMS is naturally hydrophobic with a water contact angle (WCA) of ≈110° due to its intrinsically low surface energy (21.3 mJ m^−2^).^[^ ^27^ ^]^ PDMS prefers to form conformal contact, making it extraordinarily biocompatible and highly favorable for biomedical applications. The mechanical properties of PDMS are affected by the degree of cross‐linking, and the commercial Sylgard 184 PDMS usually possesses Young's modulus of 1–3 MPa, the tensile strength of 3.5–7.7 MPa,^[^ ^28^ ^]^ and an elongation at break up to 210%. PDMS is also equipped with unique thermal properties, including one of the lowest glass transition temperatures (T_g_ ≈ −123 °C), one of the highest thermal expansion coefficients (CTE) among polymers, good heat resistance (thermally stable at temperatures below 400 °C) and low thermal conductivity (∼0.2 W m^−1^ K^−1^) owing to the flexibility of the siloxane backbone and high Si‐O bond dissociation energy of 460 kJ mol^−1^.^[^ ^29^, ^30^, ^31^ ^]^ In addition, PDMS is an electrical insulator with a low electrical conductivity of ≈10^−14^ S m^−1^ and a high breakdown voltage of 2 × 10^7^ V m^−1^.^[^ ^17^ ^]^ Furthermore, PDMS is optically transparent between 240 and 1100 nm and is permeable to certain gases.

**Table 1:** Comprehensive collection of the physical properties of Sylgard 184‐like PDMS.

Physical property	Value	Reported year
Electrical conductivity	10^−14^ S m^−1^	2016^[^ ^50^ ^]^
Breakdown voltage	2 × 10^7^ V m^−1^	2018^[^ ^17^ ^]^
Thermal conductivity	0.2 W m^−1^ K^−1^	2021^[^ ^30^ ^]^
Coefficient of thermal expansion	≈9 × 10^−14^ K^−1^ (Volumetric)	2021^[^ ^293^ ^]^
Glass transition temperature	−123 °C	2020^[^ ^29^ ^]^
Refractive index	≈1.4 at 589–1554 nm	2018^[^ ^17^ ^]^
Young's modulus	1–3 MPa	2014^[^ ^28^, ^343^ ^]^
Compressive modulus	118–187 MPa	2014^[^ ^28^, ^343^ ^]^
Elongation at break	210%	2017^[^ ^344^ ^]^
Stiffness	≈0.4 kJ m^−12^	2021^[^ ^103^ ^]^
Tensile strength	3.5–7.7 MPa	2014^[^ ^28^, ^343^ ^]^
Compressive strength	28.4–51.7 GPa	2014^[^ ^28^, ^343^ ^]^
Surface energy	21.3 mJ m^−12^	2020^[^ ^27^ ^]^
Density	0.971 g cm^−13^	2007^[^ ^29^ ^]^
Diffusion coefficient (Oxygen)	∼2000–4000 µm^2^ s^−1^	2012^[^ ^345^ ^]^
Diffusion coefficient (CO_2_)	∼1000 µm^2^ s^−11^	2015^[^ ^346^ ^]^
Diffusion coefficient (Water)	∼1000–6000 µm^2^ s^−11^	2007^[^ ^347^ ^]^
WCA	≈110°	2016^[^ ^348^ ^]^

The molding ability and tendency to seal other flat surfaces without adhesives make PDMS indispensable for molding applications, soft lithography, soft robotics, microfluidics, and flexible substrates. Another appealing feature of PDMS is its high chemical stability and non‐toxicity. It is inert to acidic solutions (e.g., hydrofluoric acid, hydrogen peroxide, and Piranha) and certain organic solvents (e.g., hexane and methanol). When other moieties replace part of the methyl groups in PDMS, the performance of the polymer will alter to a certain extent. The difference is related to the type, nature, and substitution degree of the substituents. This establishes the foundation for polysiloxane products' variety and versatility, which is uncommon in other polymeric systems. The exceptional features discussed in this section render PDMS a key component for various applications, which will be systematically introduced in the following sections.

## 3 Latest Advances in Functional PDMS‐Based Materials

### 3.1 Bulk PDMS Composites with Specifically Designed Functionality

Although pure PDMS possesses distinct attributes, its utility as an elastomer in various fields remains limited. For instance, its subpar mechanical properties impede applications in electronic or robotic stretchable devices. Additionally, unmodified PDMS exhibits insensitivity to electrical, optical, and magnetic stimuli. The incorporation of fillers serves as an efficacious method to ontologically modify PDMS, given its straightforward preparation (merely via physical mixing) and considerable enhancement efficiency. Indeed, this strategy has been implemented for numerous elastomer systems, including natural rubber where carbon black is the preferred filler.

Zero‐dimensional (0D) fillers similar to carbon black encompass micro/nanoscale particles such as SiO_2_,^[^ ^32^ ^]^ CaCO_3_,^[^ ^32^ ^]^ and ZnO,^[^ ^33^ ^]^ which have a high specific surface area, ensuring ample interfacial contact for robust interactions and augmented reinforcement efficiency. Moreover, one‐dimensional (1D) fillers like carbon nanotubes (CNTs) and metal wires, two‐dimensional (2D) platelets such as BN nanosheets^[^ ^34^ ^]^ and 3D structures (e.g., graphene foam^[^ ^35^ ^]^) can be utilized to bolster the properties of PDMS or imbue it with novel functionalities. In the following subsections, the development of the filler material, compositing method, and the added functionality will be extensively reviewed, and the explored application will be introduced as well.

#### 3.1.1 Magnetism

**Figure 3:**
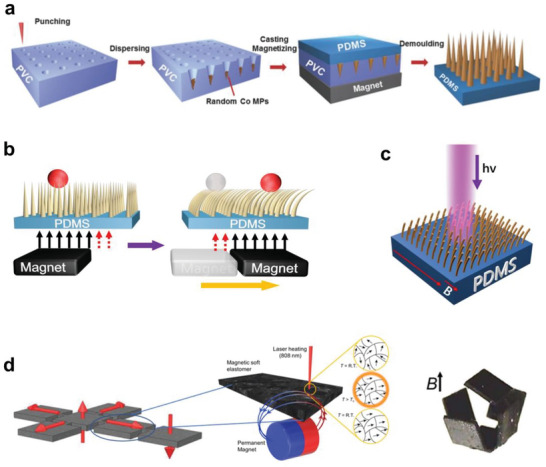
Equipping PDMS with magnetic properties. a) Fabricating flexible magnetic cilia array. Reproduced with permission.^[^ ^37^ ^]^ Copyright 2018, Wiley‐VCH. b) Oscillation mechanism of the magnetic micro cilia array under an external magnetic field.^[^ ^37^ ^]^ c) Capability of spatial resolved manipulation has been demonstrated. Locally irradiated micropillars can be partially demagnetized and are able to recover their initial vertical position.^[^ ^40^ ^]^ d) Heat‐assisted magnetic programming for deformable soft machines. Magnetic particles embedded in PDMS are locally demagnetized upon laser irradiation and then reorient under an external magnetic field. Reproduced with permission.^[^ ^41^ ^]^ Copyright 2020, American Association for the Advancement of Science.

PDMS itself is nonmagnetic. However, by adding magnetic components, the composites can be equipped with magneto‐active properties for new‐generation magnetic actuators.^[^ ^36^ ^]^ A recently explored architecture is a cilia‐inspired pillar array,^[^ ^37^, ^38^, ^39^ ^]^ which can swing back and forth in response to an external magnetic field. The hair‐like micro‐actuator array was fabricated by filling pre‐designed templates or molds with a composite paste made of PDMS prepolymer and magnetic particles, as illustrated in **Figure** **3**a. The tilting angle and speed of the pillars can be manipulated by the direction and amplitude of the applied external magnetic field. Ben et al. prepared cilia‐like elastomer arrays with cobalt magnetic particles as the key ingredient (Figure 3b),^[^ ^37^ ^]^ and they demonstrated that under alternating magnetic field modulation, nonmagnetic polystyrene (PS) microspheres could be guided to transport directionally and continuously following the motion of the flexible arrays. Compared to the columnar shape, the conically shaped array led to a higher transport efficiency. In addition, the spatial distribution of the magnetic particles within the cilia (e.g., linearly aligned or locally concentrated at the tips) can be controlled by applying a magnetic field or changing the thermal crosslinking temperature in the curing process.^[^ ^37^, ^38^ ^]^ Furthermore, various motions of the pillar array can be generated by manipulating the magnetic actuation mode. For example, Zhang et al. used the magnetic pillar array to induce a variety of flow types in a microfluidic channel, such as circulatory, direction‐reversible, oscillating, and pulsatile flows.^[^ ^38^ ^]^

It is even possible to achieve site‐selective modulation in the micro‐actuator array matrix. Figure 3c shows micropillars of magnetic CrO_2_ particles mixed with PDMS were prepared with a height–diameter aspect ratio of 8.2 and the susceptibility to force‐induced deformation.^[^ ^40^ ^]^ The light was used as a local stimulus (by a photothermal effect), which added another degree of freedom due to the low Curie temperature of CrO_2_ (395 K) and the high optical absorption of the black composite in the visible range (> 97%). Localized light‐induced demagnetization was successfully realized, demonstrating an excellent case of remote and precise local actuation in a PDMS‐based microstructural matrix.

Heat‐assisted magnetic programming can be utilized to build advanced and complex shape‐morphing magnetic soft machines. Alapan et al. reported a high‐throughput magnetic programming strategy (Figure 3d) based on heating magnetic soft materials above the Curie temperature and reorienting their magnetic domains via applied magnetic fields during cooling.^[^ ^41^ ^]^ Through laser‐assisted thermal magnetization, shape‐changing instructions in 3D can be encoded discretely and reprogrammed on‐demand with high resolution (≈38 µm). Taking advantage of magnetic re‐programmability, reconfigurable mechanical behavior of an auxetic metamaterial structure, tunable locomotion patterns of a quadrupedal soft robot, and adaptive grasping behavior of a soft gripper was achieved, respectively.

Besides mechanical actuators, magnetic elastomers can play a vital role in other types of smart devices. The addition of conductive components (such as CNTs and liquid metals (LMs)) facilitates the broad application of magnetic elastomers in flexible and wearable electronics like magneto‐resistive strain sensors^[^ ^42^, ^43^ ^]^ and flexible electromagnetic‐triboelectric nanogenerators (TENGs).^[^ ^44^, ^45^ ^]^ With the help of sugar templates, magnetic PDMS sponges can be prepared for on‐demand drug delivery and localized medical treatment.^[^ ^46^ ^]^

#### 3.1.2 Electrical Conductivity

**Figure 4:**
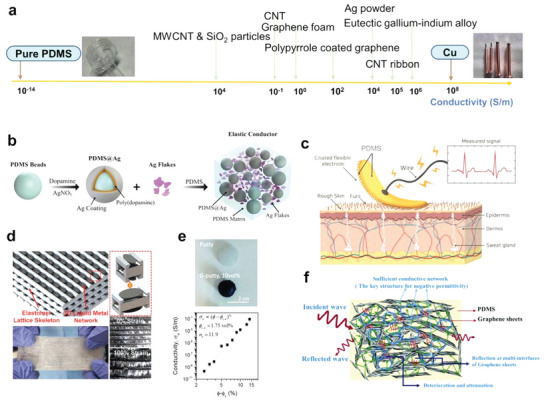
PDMS composites with various electrical conductivity. a) PDMS composites can be endowed with a wide range of conductivities by varying filler materials. b) Squeezing the PDMS beads encapsulated by silver establishes conductive paths between silver flakes. Reproduced with permission.^[^ ^49^ ^]^ Copyright 2019, American Chemical Society. c) Force‐induced bending of the microcilia array electrodes prepared from a mixture of PDMS and silver particles increases the contact area with the skin, which fills the void between the electrodes and the skin, leading to an exponential decay of resistivity. Reproduced with permission.^[^ ^52^ ^]^ Copyright 2018, Wiley‐VCH. d) EGaIn forms a continuous conductive network in the PDMS frame, and the conductivity remains stable at 0–100% strain owing to the protection of the frame. Reproduced with permission.^[^ ^54^ ^]^ Copyright 2021, Wiley‐VCH. e) Photograph of hand‐rolled spheres of putty and G‐putty. With the increase of graphene content, the conductivity of G‐putty can be increased to 0.1 S m^−1^. Reproduced with permission.^[^ ^55^ ^]^ Copyright 2016, American Association for the Advancement of Science. f) The graphene aerogel conductive backbone substantially improves the conductivity of PDMS composites, while the high porosity and the enhancement of free electrons confer electromagnetic shielding properties to the composites. Reproduced with permission.^[^ ^63^ ^]^ Copyright 2020, Royal Society of Chemistry.

PDMS is intrinsically electrical insulating with an electrical conductivity of ≈10^−14^ S m^−1^. Adding conductive fillers such as metals, LMs, and carbon‐based materials can endow PDMS composites with widely tunable conductivities, as summarized in **Table** **2** and featured in **Figure** **4**a. Elastic conductors are particularly intriguing and promising for high‐performance flexible electronics with remarkable stretchability, adhesiveness, and tunable sensitivity.

**Table 2:** Conductivity of PDMS composites with various fillers.

Electrically conductive filler	Concentration (wt% or vol%)	Mixing method	Conductivity (S m^−11^)	Reported year	Note
Ni microwires	1.2 vol%	physical blending	8 × 10^−11^	2021^[^ ^51^ ^]^	Solidification under a magnetic field to promote alignment
Silver NWs	25 wt%	xylene assisted mixing	5.8 × 10^5^	2021^[^ ^50^, ^349^ ^]^	NW length: 50 nm
Dendrite‐structured Ag powders	70 wt%	physical blending	2.7 × 10^4^	2018^[^ ^52^ ^]^	
Silver flakes	21 vol%	physical blending	1.2 × 10^5^	2019^[^ ^49^ ^]^	Flake size: 2–5 µm
Silver flakes/Ag‐coated PDMS beads	10 vol%	physical blending	1.2 × 10^5^	2019^[^ ^49^ ^]^	
Eutectic gallium‐indium alloy	50 wt%	stepwise printing	1.98 × 10^6^	2021^[^ ^54^ ^]^	
Graphene nanosheets	15 vol%	chloroform assisted mixing	1.0 × 10^−11^	2016^[^ ^55^ ^]^	Low molecular‐weight PDMS crosslinked by boric acid
Polypyrrole NWs on graphene		dip coating	5.2 × 10^2^	2021^[^ ^71^ ^]^	
PU‐Cu/Ag network		dip coating	1.5 × 10^3^	2013^[^ ^63^ ^]^	
Graphene honeycomb		dip coating	7.2 × 10	2018^[^ ^65^ ^]^	
Graphene aerogel		dip coating	1.9 × 10	2020^[^ ^66^ ^]^	Concentration of graphene:12.5 mg/ml
Graphene foam	0.7 wt%	dip coating	9.1 × 10^−11^	2020^[^ ^35^ ^]^	
Graphene foam	0.5 wt%	dip coating	10	2011^[^ ^64^ ^]^	
Multi‐walled CNTs & Silicon dioxide micro‐particles		chloroform assisted mixing	6.1 × 10^−14^	2019^[^ ^60^ ^]^	Diameter of silicon dioxide micro‐particles: 85 µm
CNT (diameter 15 nm)	5 wt%	toluene assisted mixing	1.0 × 10^−11^	2012^[^ ^350^ ^]^	
Cellulose aerogels	2.24 wt%	dip coating	47	2021^[^ ^30^ ^]^	
Graphene oxide‐coated cellulose aerogels	3.05 wt%	dip coating	75	2021^[^ ^30^ ^]^	

Silver is a highly conductive metal with an intrinsic conductivity of 6.3 × 10^7^ S m^−1^.^[^ ^47^ ^]^ Including 25 vol% Ag powder with an average size of 3 µm in PDMS results in a conductivity of 6.0 × 10^4^ S m^−1^.^[^ ^48^ ^]^ Both the distribution condition of the filler units and the interfacial connection are critical to electrical performance. For PDMS composites containing silver flakes ranging from 2 to 5 µm, the additional incorporation of silver‐coated PDMS beads (PDMS@Ag, ≈20 µm in diameter) was found to lock the position of the silver flakes in the gaps between microspheres and the dense conductive networks (Figure 4b) and improve the conductivity (surpassing 10^3^ S m^−1^) by 139 times.^[^ ^49^ ^]^

Metal nanowires (NMs) or nanorods with high aspect ratios can entangle to form conductive networks. Karimi et al.^[^ ^50^ ^]^ simply mixed ≈50 nm long silver NW with PDMS prepolymer in xylene, and after solvent removal and thermal crosslinking, they successfully fabricated a conductive film (5.8 × 10^5^ S m^−1^ with 25 wt% silver). Promoting the directional arrangement of linear fillers can further improve anisotropic electrical conductivity. Magnetic field‐assisted al/.=o][l..ignment of nickel microwires with a length‐to‐diameter ratio of 150:1 was realized in a PDMS film during the crosslinking process, increasing the conductivity to 0.8 S m^−1^ with only 1.2 vol% of Ni^[^ ^51^ ^]^; this material can be applied toward the development of highly sensitive flexible piezoresistive pressure sensors.

Conductive electrodes with 3D microstructures were also explored for advanced electromechanical applications. Jin et al.^[^ ^52^ ^]^ cured PDMS prepolymers doped with dendritic silver particles in a polymethyl methacrylate (PMMA) mold to prepare microcilia array electrodes with a conductivity of 2.7 × 10^4^ S m^−1^. As illustrated in Figure 4c, under external pressure, the cilia bent to increase the contact surface area between the electrode and the skin, resulting in an exponential decay of the resistivity. This structure allows the accurate measurement of the bioelectrical signals on rough skin without compromising the electrode's performance.

LMs, such as gallium‐based eutectic alloys, possess not only high conductivity but also excellent processability and good fluidity, and are perfect fillers for stretchable conductors. However, LMs also experience high surface tension with a thin oxide skin (≈1–3 nm in thickness) spontaneously formed upon exposure to air,^[^ ^53^ ^]^ resulting in the parasite separate blocks in the elastomer matrix, which hinders a continuous conductive path. Wang et al. invented a stepwise 3D printing approach to fill the open cells of the PDMS framework with a gallium‐indium alloy (EGaIn) to impart a 3D continuous conductive network with conductivity as high as 1.98 × 10^6^ S m^−1^ (with 50 wt% of EGaIn).^[^ ^54^ ^]^ One invaluable finding is that this composite was electrically invariant, showing only 2% resistance change across a wide strain range from 0 to 100% (Figure 4d). Surprisingly, the EGaIn content had a negligible impact on the mechanical properties of the composites, avoiding the conventionally observed impairment caused by severe filler agglomeration.

Carbon nanomaterials, such as CNTs and graphene, have been intensively investigated as nanofillers in PDMS for different purposes. With the latest advances in these carbon nanomaterials, this field has enormous possibilities. Advanced methods for fabricating PDMS composites have been developed to produce various functional elastic conductors. Boland et al. mixed graphene nanosheets with viscoelastic polymer putty (low molecular‐weight PDMS lightly crosslinked by boric acid) in chloroform to obtain a high‐performance electromechanical material, G‐putty (Figure 4e),^[^ ^55^ ^]^ which exhibited unusual and exceptional electromechanical behaviors, such as post‐deformation temporal relaxation of electrical resistance and non‐monotonic changes in resistivity with strain. These extraordinary features are related to the mobility and connectivity of the nanosheets in the low‐viscosity polymer matrix. The electrical conductivity of the G‐putty increased with graphene content, reaching ≈0.1 S m^−1^ at ≈15 vol%. G‐putty is also a highly sensitive electromechanical sensor with gauge factors > 500 in a sample fabricated with a 6.8 vol% of graphene nanosheet content and can measure pulse, blood pressure, and even the footsteps of tiny spiders.

CNTs have long been considered essential fillers for flexible conductive composites due to their extraordinary electrical conductivity and high aspect ratio.^[^ ^56^ ^]^ Still, the uncontrollable distribution of CNTs in the matrix often severely jeopardizes the performance of PDMS composites. It is also important to note that CNTs represent an extraordinarily rich and diverse class of materials featuring vastly different structures and thus physical properties.^[^ ^57^, ^58^, ^59^ ^]^ Judicious choices of specific CNT types are critical to understand the literature and harnessing their remarkable properties in intended applications.

To overcome the CNT dispersion challenge in PDMS, Chen et al. introduced SiO_2_ particles into the PDMS‐CNT matrix for a highly sensitive piezoresistive sensor and observed a fivefold increase in conductivity because the SiO_2_ particles exhibited a volume exclusion effect on the dense CNTs filler material.^[^ ^60^ ^]^ SiO_2_ particles with high modulus values transfer stress to the PDMS/CNT phase, resulting in higher sensitivity. A recently explored architecture is a highly porous PDMS‐CNT foam synthesized via a sacrificial sugar template.^[^ ^61^, ^62^ ^]^ The resultant material was highly sensitive to the applied external force, with a response time of milliseconds and a detection limit as low as 0.077% in strain or 0.18 Pa in vertical pressure.

Composites prepared by a conventional physical blending method often show unsatisfactory performance, primarily owing to unwanted filler aggregation, unstable conductive networks, and the significantly reduced stretchability of composites at high filler loading. Zheng et al. developed a strategy using a 3D conductive network as the filler in the elastomer to produce conductive composites. They deposited metal flakes (Cu, Ag, and Au) on a chemically functionalized porous polyurethane (PU) sponge to form a continuous 3D conductive network and mixed it with PDMS to obtain PU‐metal‐PDMS conductive composites (Figure 4f). The sponge structure acts as a strain buffer, that prevents crack formation. Interestingly, the PU‐metal‐PDMS composites were patternable by printing methods, which is crucial for their application as interconnects.^[^ ^63^ ^]^ The 3D conductive network approach has been further explored recently. Graphene foam has been frequently used to construct elastic conductors due to its 3D interconnected structure. The composite exhibited a conductivity of 0.9 S m^−1^ at a relatively low graphene loading of 0.7 wt%,^[^ ^35^ ^]^ and 10 S m^−1^ with a higher loading of 5 wt%.^[^ ^64^ ^]^ Additionally, composites with such architectures can attain high stretchability while maintaining stable conductivity through the design of macroscopic and microscopic continuous carbon networks. The superior mechanical flexibility of the honeycomb‐like graphene induced an unchanged conductivity of 72 S m^−1^ under the strain range of 0%–60%.^[^ ^65^ ^]^ Ni et al. successfully prepared a conductive film with an electrical conductivity of 19 S m^−1^ by sealing a multi‐interfaced graphene aerogel in PDMS^[^ ^66^ ^]^ and tested the as‐synthesized composite for its performance in electromagnetic interference shielding applications. The overall shielding effectiveness is as high as 60 dB, which is attributable to the graphene aerogel's high porosity and the improved reflection of electromagnetic waves resulting from enhanced free electron density.

#### 3.1.3 Thermal Properties

PDMS‐based composites are promising candidates for thermal interface materials due to the high mechanical flexibility, electrical insulation, and chemical inertness of PDMS.^[^ ^67^ ^]^ However, the intrinsically low thermal conductivity of PDMS (≈0.15 W m^−1^ K^−1^)^[^ ^68^ ^]^ may prevent its use in high‐performance applications. To overcome this limitation, thermally conductive fillers are required. As summarized in **Table** **3**, a variety of thermally conductive fillers (such as metal, metal oxide, CNT, graphene, and boron nitride (BN)) have been incorporated in the PDMS matrix to tune its thermal conductivity.

**Table 3:** Thermal properties of PDMS composites with various fillers.

Thermally conductive filler	Concentration (wt% or vol%)	Thermal conductivity (W m^−11^ K^−11^)	Increased thermal stable temperature ΔTd (°C)	Reported year
Al	14 vol%	9 × 10^−11^		2021^[^ ^69^ ^]^
3D Al	14 vol%	1.6		2021^[^ ^69^ ^]^
Al_2_O_3_‐ZnO NWs	40 vol%	1.8	270	2020^[^ ^33^ ^]^
Polypyrrole NWs anchor graphene		4.8 × 10^−11^		2021^[^ ^71^ ^]^
Nickel‐coated carbon fibers	51.54 wt%	1.1 × 10		2021^[^ ^68^ ^]^
Highly oriented graphite	17.6 vol%	3.5 × 10		2022^[^ ^73^ ^]^
Cellulose carbon aerogel@rGO	3.05 wt%	6.5 × 10^−11^	30	2021^[^ ^30^ ^]^
Silica‐coated graphene nanoplatelet	2 wt%	4.9 × 10^−11^		2019^[^ ^72^ ^]^
Cellulose nanocrystals	4 wt%		140	2016^[^ ^351^ ^]^
Polyethylene microfibers	55 wt%	3.8 × 10		2021^[^ ^352^ ^]^
BN nanosheets	35 wt%	1.2		2020^[^ ^34^ ^]^
Sphere BN	35 wt%	7.7 × 10^−11^		2020^[^ ^34^ ^]^
BN nanosheet‐cellulose nanocrystal		7.3		2021^[^ ^75^ ^]^
BN aerogel	25.4 wt%	1.6		2020^[^ ^74^ ^]^
Silica aerogel	12 wt%	1.8 × 10^−12^		2019^[^ ^77^ ^]^
Carbon Fibers	0.5 wt%		25	2021^[^ ^353^ ^]^
CNT (multi‐walled)	5 wt%	8.2 × 10^−11^	9	2016^[^ ^354^ ^]^

Traditional metal fillers can impart high thermal conductivity but they may also result in undesired electrical conductivity that hampers the electronic systems. For such applications fillers that are thermally conductive while electrically insulating are highly desirable. Wei et al. sealed a 10 nm‐thick SiO_2_ layer‐coated 3D continuous Al framework in PDMS,^[^ ^69^ ^]^ forming a composite that was electrically insulative while attaining a high thermal conductivity of 1.6 W m^−1^ K^−1^. Ceramic materials such as Al_2_O_3_ have been widely employed to fabricate thermally conductive and electrically insulative composites, but high filler loading is required to construct a continuous phonon transport path. Liu et al. designed a “hairy sphere” structure (Al_2_O_3_ spheres with ZnO NWs grown perpendicularly to the surface) as a novel hybrid filler.^[^ ^33^ ^]^ The ZnO NWs reduced the interfacial thermal resistance between the Al_2_O_3_ spheres and PDMS matrix, eliciting a high thermal conductivity of ≈1.2 W m^−1^ K^−1^ with a filler content of 40 vol%.

Graphene has attracted much attention as a promising thermal conductive filler owing to its high thermal conductivity (≈5300 W m^−1^ K^−1^) and lightweight.^[^ ^70^ ^]^ Surface modification of the graphene filler has been explored to tune the composites' conductivity and interfacial thermal resistance. Zhang et al. deposited polypyrrole NWs on a graphene surface with an electrochemical polymerization method and found that the nanowires deeply penetrated the PDMS matrix after compounding, reducing the interfacial thermal resistance.^[^ ^71^ ^]^ SiO_2_ shows excellent compatibility and wettability with PDMS; therefore, a SiO_2_ coating layer can facilitate the interaction between graphene and PDMS and effectively prevent agglomeration.^[^ ^72^ ^]^ A 3D carbon network can be built to provide a continuous thermal conductive path. Zhang et al.^[^ ^73^ ^]^ reported a facile, one‐step liquid expansion method to construct a directional graphene network, achieving a high cross‐surface thermal conductivity of 35.4 W m^−1^ K^−1^ with a graphene content of 17.6 vol%.

Novel filler materials have also been explored recently. BN nanosheets, which are electrically insulative and highly thermal conductive (1700–2000 W m,^−1^ K^−1^) were incorporated via direct mixing for efficient heat dissipation.^[^ ^34^ ^]^ In addition, BN aerogels were integrated with PDMS to generate a composite with high thermal conductivity and low electrical conductivity of 10^−13^ S m^−1^.^[^ ^74^ ^]^ Recent experiments demonstrated that the device based on a sandwich structure with the BN‐cellulose nanocrystal mixture in the middle of two PDMS layers managed to cool down the surface of a ceramic heating plate from 105 to 76 °C within 60 s.^[^ ^75^ ^]^

The PDMS composite can be used for thermal insulation as well. Pores in the composite significantly reduce the thermal conductivity ascribed to the ultralow thermal conductivity of the entrapped air (≈0.026 W m^−1^ K^−1^).^[^ ^76^ ^]^ Lee et al. fabricated a flame‐retardant, porous PDMS‐silica aerogel composite with a thermal conductivity as low as 0.018 W m^−1^ K^−1^ after removing the ethanol that was pre‐packed in the gap of the silica aerogel.^[^ ^77^ ^]^ Zhou et al. reported a porous PDMS sponge prepared with a recyclable micro‐sugar template for passive radiative cooling.^[^ ^78^ ^]^ The synthesized white sponge exhibits strong visible light scattering and thermal emission, demonstrating a cooling power of 43 W m^−2^ and temperature drop of 4.6 °C under 1 sun irradiation. Furthermore, the air‐filled voids within the PDMS sponge suppressed the overall thermal conductivity to 0.06 W m^−1^ K^−1^. The combination of the PDMS sponge's radiative cooling and thermal insulation properties enables smart heat‐regulation and promising energy‐saving applications.

#### 3.1.4 Optical Features

PDMS is optically transparent at 240–1100 nm and can serve as a flexible substrate or carrier for various light‐absorbing and luminescent materials, allowing the fillers to define the optical properties of the composite. These composites have demonstrated practical applications within the areas of smart displays,^[^ ^79^ ^]^ camouflage,^[^ ^80^ ^]^ anti‐counterfeiting,^[^ ^81^ ^]^ wearable light‐emitting devices,^[^ ^82^ ^]^ and various sensors.^[^ ^83^ ^]^ For example, the surface plasmonic resonance of metal nanoparticles with controllable size can generate precisely tunable absorption characteristics. Au@SiO_2_ nanoparticles were dispersed in PDMS for fabricating eyeglass lenses with tunable and narrow absorption bands, which can be customized for patients with color vision deficiency at specific wave bands.^[^ ^84^ ^]^

**Figure 5:**
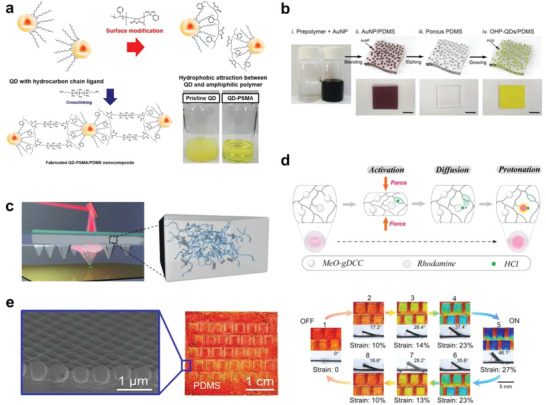
PDMS with diverse optical properties. a) Surface‐modified QDs act as a crosslinker for PDMS owing to the ring‐opening reaction between maleic anhydride and diamines. Reproduced with permission.^[^ ^88^ ^]^ Copyright 2020, Elsevier. b) Schematic of the synthesis of the organometallic halide perovskite quantum dots (OHP‐QDs) well dispersed in PDMS. Scale bar = 1 cm. Reproduced with permission.^[^ ^92^ ^]^ Copyright 2020, Royal Society of Chemistry. c) Photoactuated polymer pens made of PDMS and surface functionalized short CNTs. Due to the superior photothermal properties of CNTs and the high CTE of PDMS, the localized photoactuation enables the dynamic control of polymer pens for molecular printing. Image on the left‐hand side: Reproduced with permission.^[^ ^96^ ^]^ Copyright 2018, Wiley‐VCH. Image on the right‐hand side: Reproduced with permission.^[^ ^22^ ^]^ Copyright 2023, Multidisciplinary Digital Publishing Institute. d) Schematic of PDMS grafted by MeO‐gDCC for mechanochromism and chemical signaling. The film turns pink due to the bimolecular chromogenic reaction between the released HCl and the rhodamine dye. Reproduced with permission.^[^ ^98^ ^]^ Copyright 2020, American Chemical Society. e) Mechanically triggered color switchable photonic crystal PDMS kirigami. States 1–5 are the tensioning process in which the gate is raised and the color changes from red to blue. Conversely, states 5–8 are the unloading process, in which the color returns to the same value as in the loading process. Reproduced with permission.^[^ ^80^ ^]^ Copyright 2021, Wiley‐VCH.

Quantum dots (QDs) have attracted significant attention owing to their optical tunability and solution processability,^[^ ^85^ ^]^ and can contribute to flexible luminescent devices when coupled with PDMS. To prevent particle aggregation and performance degradation,^[^ ^86^, ^87^ ^]^ new approaches have been executed to improve the dispersion of the QDs. An amphiphilic polymer, poly(styrene‐co‐maleic anhydride), was used to encapsulate CdSe@ZnS/ZnS core/shell QDs and further crosslink them with amine‐terminated PDMS at a low curing temperature owing to the ring‐opening reaction between maleic anhydride and the diamines (**Figure** **5**a). The resulting composite demonstrated the uniform distribution of QDs and high transparency even at a high QD concentration (30 wt%) and was used to fabricate a light‐emitting diode device with impressive efficiency.^[^ ^88^ ^]^

Metal halide perovskites (APbX_3_, X = Cl, Br, and I) have attracted great interest owing to their extraordinary optical properties;^[^ ^89^, ^90^ ^]^ however, critical problems remain regarding size uniformity and long‐term stability. The marriage between PDMS and perovskite QDs has opened the door to new solutions. PDMS can be used to fabricate soft templates to confine the size and position of the perovskite nanocrystals during the growth process.^[^ ^91^ ^]^ Cha et al. created a porous template by etching Au nanoparticles with a specific size that had been previously embedded in the PDMS film.^[^ ^92^ ^]^ The template was then immersed in a methylammonium lead trihalide (MAPbX_3_) precursor solution for multiple times for the full growth of MAPbX_3_ QDs in pores (Figure 5b). The pores defined the size of QDs, prevented aggregation, and kept the QDs away from the ambient environment. Consequently, the photoluminescence (PL) peak of the QDs shifted as the size of the original Au nanoparticles varied, and the PL quantum yield remained stable even after seven months. Encapsulated liquid PDMS prepolymers affect the growth of the perovskite nanocrystals as well. Halide perovskite inks were inkjet‐printed into liquid PDMS prepolymer,^[^ ^86^ ^]^ which significantly retarded the crystallization process by prohibiting contact with air and reducing the solvent evaporation rate. The obtained fluorescent single crystals demonstrated that the simple inkjet printing method can produce wafer‐scale, air‐stable, and flexible perovskite fluorescent patterns. In addition, PDMS can be utilized as a gas‐permeable and diffusive flexible host to control a room‐temperature anion exchange reaction for embedded CsPbBr_3_ nanocrystals, providing a tunable halide component in the matrix.^[^ ^93^ ^]^

Chemical composition and surface moiety modification effectively enhance the optical property of the perovskite QDs and their dispersion in PDMS. Gong et al. adopted an organosilicon (3‐aminopropyltriethoxy‐silane‐glutaric anhydride) ligand to cap CsPbBr_3_ QDs, which enhanced the thermal stability of QDs and improved the compatibility and cohesion between brittle QDs and the flexible polymer matrix owing to the similar structure of PDMS and the ligands.^[^ ^94^ ^]^ After heat treatment at an elevated temperature (125 °C), the composites still achieved 95.5% PL quantum yields. Strikingly, PL enhancement occurred during material elongation, which was interpreted as the elimination of emission reabsorption among QDs. Wang et al. dissolved Mn^2+^ doped perovskite QDs (CsPbX_3_: Mn) in cyclohexane and PDMS prepolymer to form fluorescent inks that could be used to print fluorescent patterns on different flexible substrates. The printed patterns showed bright red, green, and cyan colors that remained bright after 60 days due to the optical and thermal stability induced by Mn^2+^ doping and the encapsulating effect by PDMS.^[^ ^95^ ^]^

Optically tailored PDMS composites can enable diverse applications, including actuators and sensors. Photoactuation is especially preferable for inducing local mechanical work with high spatiotemporal precision and resolution due to its intrinsic advantages, which include remote control, wireless access, and spatial selectivity. Huang, Wang, and co‐workers developed photoactuated polymer pens (Figure 5c) made of PDMS and surface‐functionalized short CNTs for molecular printing.^[^ ^22^, ^96^ ^]^ The surface grafting of long alkyl chains on CNT walls facilitated the dispersion of these carbon nanomaterials in the PDMS matrix, producing a highly light‐absorbing yet transparent composite nearly free of light scattering. Localized photoactuation of selected polymer pens, which was directed by a digital micromirror device, created an out‐of‐plane motion by more than 7 µm adequate for active molecular printing. This strong photoactuation effect could not be achieved without the superior photothermal properties of CNTs and the high CTE of PDMS.

A chemical sensor, the PDMS‐ZnO QDs‐based fluorescent mouthguard, was invented to accurately locate dental lesion sites.^[^ ^97^ ^]^ The chemical reaction between ZnO and volatile sulfur compounds (such as H_2_S) released by normal dental caries quenched the fluorescence locally in the composite, showing a 40–50% decline in intensity between 550 and 570 nm within 7 min.

Mechanochromic devices are designed and fabricated based on a stain‐triggered color change. Lin et al. designed a novel mechanophore for mechanochromic response.^[^ ^98^ ^]^ The mechanoacid 2‐methoxy‐substituted gem‐dichlorocyclopropane (MeO‐gDCC) generates HCl in a mechanochemical ring‐opening reaction (Figure 5d) with a transition force as low as 880 pN. Based on this chemistry, Lin et al. incorporated multiple segments of MeO‐gDCC into the PDMS polymer backbone to produce a clear, pale pink elastomer. The film turned pink when it was exposed to tensile strain, compression, or localized compression due to the bimolecular chromogenic reaction between the released HCl and the rhodamine dye. Since the response is slow and time‐dependent, it is possible to trace back to the initial starting time by extrapolating an apparent activation point from the late‐time behavior.^[^ ^98^ ^]^

Advanced approaches have also been developed to build flexible mechanoluminescence (ML) devices. Since a soft and flexible elastomer matrix might act as a stress buffer and decrease the sensitivity, SiO_2_ nanoparticles were included in the hybrid ink to concentrate stress in ZnS:M^2+^(Mn/Cu)@Al_2_O_3_ micro‐sized particles.^[^ ^82^ ^]^ The intense ML, ascribed to the piezoelectrically induced electron detrapping electrofluorescence in doped ZnS, was realized under weak stimulation (strain = 5–30%). The printed SiO_2_ nanoparticle‐doped composite matrix film is compatible with the elasticity modulus of human skin and can achieve skin‐driven ML, enabling the presentation of fetching augmented animated expressions.

Structural color, based on the dynamic scattering of light by micro‐ or nano‐structures, has been found in birds, butterflies, and plants, and continues to fascinate scientists with its high tunability and enduring photonic properties.^[^ ^83^ ^]^ Inspired by the color‐switching modulation ability found in nature, Lai et al. created a mechanically triggered color‐switchable elastomer kirigami with unprecedented repeatability (recyclability >10^4^), by fabricating an array of rectangular cuts on a thin PDMS film and then firmly bonding the mono‐dispersed PS spheres onto the PDMS surface. The 2D photonic crystal array presented a color variation across the entire visible spectrum from violet to red by varying the view angle (see Figure 5e), and the out‐of‐plane pop‐up angle of the kirigami could be controlled by in‐plane stretching in a programmable manner.^[^ ^80^ ^]^ This research is critical for advancing practical applications such as cryptography, sensor technology, dynamic displays, and camouflage.

Phase transition materials can be coupled with PDMS for a temperature‐induced smart display. The elastic PDMS serves as a compatible host for vapor, liquids, or solids. Paraffin is a mixture of hydrocarbons with ≈18–30 carbons and melts at ≈45–55 °C.^[^ ^99^, ^100^ ^]^ The PDMS‐paraffin composite film enabled reversible switching between a transparent and opaque surface under a thermal stimulus.^[^ ^101^ ^]^ The solid‐to‐liquid phase transition of paraffin enhanced the average transmittance of the composite film to > 80% between 400 and 800 nm,^[^ ^102^ ^]^ and the composite holds promise for use in smart windows.

#### 3.1.5 Mechanical Properties

**Figure 6:**
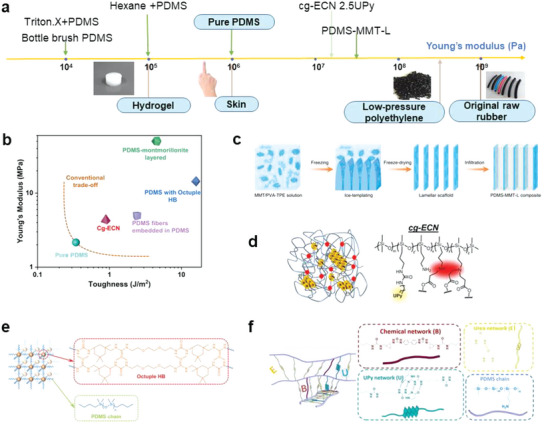
Strategies to mechanically strengthen PDMS. a) Young's modulus of PDMS can be manipulated across a wide range. b) Summary of PDMS composites that have achieved a significant increase in both Young's modulus and toughness values simultaneously. c) Preparation process of the nacre‐inspired PDMS‐montmorillonite layered nanocomposites. The continuous scaffold in PDMS can bear high loads and prevent crack extension. Reproduced with permission.^[^ ^103^ ^]^ Copyright 2021, Nature Publishing Group. d) Mastering hydrogen‐bonding clusters (red parts in polymer networks) to simultaneously improve cg‐ECN's stiffness and extensibility. Reproduced with permission.^[^ ^104^ ^]^ Copyright 2022, Cell Press. e) Homogeneous, strong, reversible network consisting of PDMS chains (blue) and octuple hydrogen bonds. Reproduced with permission.^[^ ^105^ ^]^ Copyright 2022, Wiley‐VCH. f) PDMS‐based high‐strength elastomer designed with a high‐density network structure combining permanent chemical bonding and dynamic physical bonding, B represents benzi‐diNCO, U represents UPy‐NCO, and E represents e‐NCO. Reproduced with permission.^[^ ^106^ ^]^ Copyright 2023, Cell Press.

As an elastomer, PDMS is known for its excellent flexibility and stability. However, its crack resistance, or toughness, is relatively poor. The mechanical properties of pure PDMS are usually controlled by the degree of crosslinking present in the polymer. Adjusting the degree of crosslinking can effectively tune the polymer's Young's modulus (i.e., 0.3 to 3 MPa). However, strong crosslinking may also lead to the impairment of PDMS's stretchability. Conventional wisdom believes that there usually exists a tradeoff between stiffness and extensibility/toughness for covalently crosslinked polymers. Nevertheless, the latest advances in materials science, compounding methods, and modification chemistry provide exciting opportunities to tune the mechanical properties of elastomer composites at a much wider range (**Figure** **6**a) and successfully move past these aforementioned trade‐offs (Figure 6b).

Inspired by nacre, Cheng and Tang et al. prepared PDMS‐montmorillonite layered nanocomposites via ice‐templating, which led to a 23‐ and 12‐fold enhancement in Young's modulus and toughness, respectively.^[^ ^103^ ^]^ Montmorillonite, a natural clay, was exfoliated into nanosheets and blended with aggregation‐induced emission (AIE) luminogens‐decorated polyvinyl alcohol to form a homogeneous solution, followed by bidirectional freezing, during which ice crystals formed parallel platelets and generated a lamellar structure (Figure 6c). After the sublimation of the ice, PDMS was eventually introduced into the lamellar scaffold to fill the voids. The authors used AIE‐assisted confocal fluorescence microscopy to observe the crack propagation and probe the stiffening and toughening mechanisms. The researchers determined that the substantial increase in the value of Young's modulus arose from the continuous scaffolding, which can bear a higher load at the beginning of the stretching, and that the enhanced toughness stemmed from the crack deflection and crack bridging in the layered structure, preventing the rapid transverse propagation of the crack.

Dynamic hydrogen bonding is key in manipulating the elastomer's mechanical properties. Cao et al. reported that both Young's modulus and elongation at break can be improved simultaneously via controlling the distribution, size, and topology of the incorporated hydrogen‐bonding clusters.^[^ ^104^ ^]^ Aza‐Michael addition between (aminopropylmethylsiloxane)‐dimethylsiloxane copolymer (four amines per polymer chain) and the acryloxy‐terminated ethyleneoxide dimethylsiloxane‐ethylenoxide block copolymer created an elastic chemical network (recorded as ECN), while hydrogen‐bonding units, such as 2‐ureido‐4[1H]‐pyrimidinone (UPy), were introduced through physical blending or chemical grafting on the side chains of PDMS (recorded as cg‐ECN, see Figure 6d). Both stiffness and extensibility were enhanced by 158 and 3 times, respectively, for the chemically grafted elastic chemical network, whose dynamic stretching process was discovered to contain two stages: an elastic recovery region (0–10% strain) and the breakage of chemical bonds and rearrangement of UPy clusters (10–150% strain). This study highlights the importance of the interaction between the hydrogen‐bonding pairs, along with the topology of hydrogen‐bond clusters, on the mechanical properties of PDMS composites. Similarly, by adding strong, reversible, and sacrificial octuple hydrogen bonding into linear PDMS via the chain extension reaction, Zhuo et al. built highly homogeneous and energy dissipative networks (Figure 6e) which were capable of evenly distributing the stress to each polymer chain thus, decreasing the stress concentration and delaying fracture.^[^ ^105^ ^]^ Furthermore, the synthesized elastomer exhibited distinct microphase separation, containing the soft hydrophobic PDMS segments and hard hydrophilic hydrogen bonding nanodomains. These strong hydrogen bonding nanodomains restricted the mobility of polymer chains and transformed between different configurations to dissipate energy under stress, further enhancing both stiffness and toughness.

An innovative recyclable elastic network (REN) elastomer, based on PDMS, was developed by Cao et al., featuring a high‐density network that seamlessly integrates permanent chemical bonds with dynamic physical ones. The precursor PDMS, rich in side chains (with 48 amine units), facilitated the formation of an extremely high crosslinking density network through sequential reactions with benzidine‐isophorone diisocyanate (benzi‐diNCO), ethyl isocyanate (e‐NCO), and ureido‐4[1H]‐pyrimidinone isocyanate (UPy‐NCO) (refer to Figure 6f). The incorporation of benzi‐diNCO transformed it into a robust chemical cross‐linker, enhancing the mechanical properties, while the quadruple hydrogen bonding sites of UPy create a potent physical dynamic network. Lastly, the addition of e‐NCO transformed the remaining amine into urea units, engendering another dynamic network based on hydrogen bonding. The resulting REN elastomers far exceed most of their PDMS counterparts in mechanical strength (10 MPa) and toughness (66 MJ/m^3^) as per report.^[^ ^106^ ^]^

Interfacial interaction between the filler and the matrix is critical to the overall performance of the composite. Gardea et al. created a CNT‐PDMS composite with the nanofiller‐polymer interfacial chemistry rendered responsive to UV light through photoreactive benzophenone. This light‐responsive interface allows programming the composite's elastic modulus and yield stress by exposure to the light stimulus.^[^ ^107^, ^108^ ^]^

**Figure 7:**
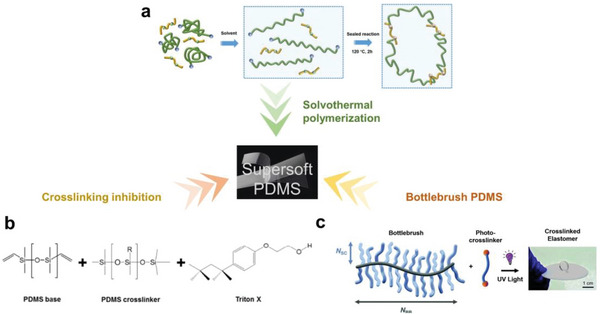
Strategies for developing supersoft PDMS. a) Solvothermal polymerization of PDMS in hexane ensures that the network of crosslinked elastomer molecules is untangled and unbroken during subsequent stretching. Reproduced with permission.^[^ ^109^ ^]^ Copyright 2021, Springer. b) Adding crosslinking inhibitor (herein, Triton‐X) to reduce the degree of crosslinking. Reproduced with permission.^[^ ^110^ ^]^ Copyright 2018, American Chemical Society. c) Synthesizing highly branched bottlebrush PDMS with minimum chain entanglement. Reproduced with permission.^[^ ^112^ ^]^ Copyright 2020, Royal Society of Chemistry Mater.

By controlling the crosslinking process, PDMS‐based composites can also be made extremely soft and super‐stretchable (**Figure** **7**). One facile but clever method is to involve the solvent in the curing process. Yu and co‐workers reported a solvothermal polymerization process with the addition of n‐hexane to change the structure of crosslinking networks; the researchers synthesized a PDMS‐based material with a maximum elongation of over 3000% (>10 times above normal values) and a tensile modulus lower than 0.15 MPa (< 1/10 of typically observed values), which can be utilized for oil collection and organic solvent sensing.^[^ ^109^ ^]^ Another approach is to manage the crosslinking speed and density by controlling the catalyst amount or the type of crosslinking chemistry. As mentioned in Section 2.1, a variety of chemical groups can be used to inhibit the crosslinking chemistry of PDMS based on the reaction between Si‐H and vinyl terminals. The polar functional groups in a widely used non‐ionic surfactant, Triton‐X, interact with the Pt catalyst, and were utilized to intentionally hinder the crosslinking and modulate the mechanical properties of the PDMS polymer.^[^ ^110^ ^]^ As a result, the soft PDMS composite film was highly adhesive, compliant, and comfortable, enabling its application in epidermal biosensors. Skov et al. discovered that the unwanted side‐reaction (oxidative crosslinking of Si‐H groups in the presence of oxygen) in the conventional formulations proceeded very slowly and allowed for the preparation of highly diverse networks using a simple one‐pot reaction.^[^ ^111^ ^]^ Furthermore, by leveraging the intrinsic “supersoft” characteristics of bottlebrush polymers, Reynolds et al. synthesized a photocurable bottlebrush PDMS, with a modulus of 10^4^–10^5^ Pa that is 10–100 times smaller than conventional linear analogs and can be used for highly sensitive capacitive pressure sensor applications affording an enhanced sensitivity (up to 53 times greater) compared to Sylgard 184.^[^ ^112^ ^]^

#### 3.1.6 Self‐Healing Capability

**Figure 8:**
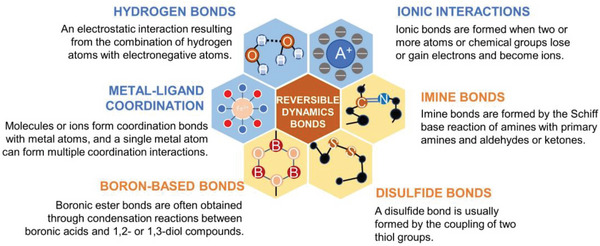
Display of a handful of reversible dynamic bonds with inherent self‐healing mechanisms, which have been successfully introduced to PDMS‐based elastomer.

Self‐healing is the capability of a material to recover from physical damage,^[^ ^113^ ^]^ and, depending upon the mechanism by which it operates, can be classified as either microencapsulated self‐healing or intrinsically self‐healing (**Figure** **8**), with the latter triggered by reversible dynamic covalent bonds (e.g., disulfide bonds, imine bonds, etc.) or non‐covalent bonds (hydrogen bonds, ionic bonds, etc.). Due to its irreversibly crosslinked structure, PDMS cannot usually recover its original form and function after sustaining damage. However, the newly developed self‐healing silicone composites (as summarized in **Table** **4**) have extended the service life of the materials and increased the types of applications into which they can be incorporated (e.g., coatings, sensors, nanogenerators, controlled‐release drugs, and sealing materials).

**Table 4:** Summary of self‐healing PDMS designed by diverse strategies.

Operation principle	Healing efficiency (condition)	Reported year	The parameter used to calculate the healing efficiency
Capsule based		2006^[^ ^115^ ^]^	tear strength
Capsule based	70–100%	2007^[^ ^117^ ^]^	tear strength
Nanofibers based		2017^[^ ^118^ ^]^	–
Ionic interactions (amino groups and the acid groups)	77%	2016^[^ ^132^ ^]^	strain
Ionic bonds	84% (4 h at 80 °C)	2020^[^ ^134^ ^]^	strain
Hydrogen bonds	100% (20 min at 40 °C)	2018^[^ ^355^ ^]^	strain
Hydrogen bonds	78% (48 h at RT)	2018^[^ ^129^ ^]^	strain
Hydrogen bonds	78% (48 h at RT)	2018^[^ ^130^ ^]^	strain
Hydrogen bonds	≈100% (12 h at 60 °C)	2019^[^ ^123^ ^]^	–
Hydrogen bonds	100% (5 h at 80 °C)	2020^[^ ^124^ ^]^	strain
Hydrogen bonds	97% (30 min at RT)	2021^[^ ^125^ ^]^	mechanical toughness
Hydrogen bonds	100% (1 h at 120 °C)	2021^[^ ^120^ ^]^	stress
Hydrogen bonds	98%–100%	2017^[^ ^119^ ^]^	mechanical toughness
Ce‐coordination bonds	80% (48 h at 60 °C)	2018^[^ ^356^ ^]^	strain
Zn‐coordination bonds	≈98% (24 h at RT)	2019^[^ ^357^ ^]^	strain
Zn‐coordination bonds	≈76%	2016^[^ ^135^ ^]^	strain
Zn‐coordination bonds	≈90% (24 h at RT)	2020^[^ ^136^ ^]^	strain
Fe‐coordination bonds	≈90%	2017^[^ ^137^ ^]^	strain
Imine bonds	94% (12 h at RT)	2018^[^ ^142^ ^]^	strain
Imine bonds	96% (6 h at 60 °C)	2021^[^ ^143^ ^]^	strain
Disulfide bonds	95% (10 h at RT)	2020^[^ ^139^ ^]^	strain
(B‐H) Covalent boroxine bonds	100% (12 h at 70 °C)	2016^[^ ^146^ ^]^	strain
B‐O bonds		2021^[^ ^147^ ^]^	–
Hydrogen bonds(healing) and Al‐coordination bonds (robustness and elasticity)	91% (36 h at RT)	2019^[^ ^358^ ^]^	integral area in tensile curve after healing divided by that of the uncut samples
Hydrogen bonds, imine bonds and Si‐O chains	95% (24 h at RT)	2020^[^ ^150^ ^]^	strain
Hydrogen bonds and disulfide bonds	93% (2 h at RT)	2020^[^ ^151^ ^]^	strain

In 2001, White et al. reported a buried microencapsulated self‐healing material.^[^ ^114^ ^]^ The capsule containing the repair agent ruptures when the material is damaged, and the released repair agent moves to the damaged site and completes the repairing reaction. White's group then applied this design to PDMS.^[^ ^115^, ^116^ ^]^ To further improve the stability of the restorative agent, Keller et al. separated the vinyl‐functionalized PDMS and Pt catalysts from the PDMS with active sites by placing them into different microcapsules. When the designed microcapsules were embedded into a PDMS matrix with a low modulus and prone to strain‐failure, the self‐healing efficiency increased to 76%.^[^ ^117^ ^]^ Furthermore, nanofibers (polyacrylonitrile fiber) can be used to store the PDMS matrix (repair agents) to impart the self‐healing capability.^[^ ^118^ ^]^

**Figure 9:**
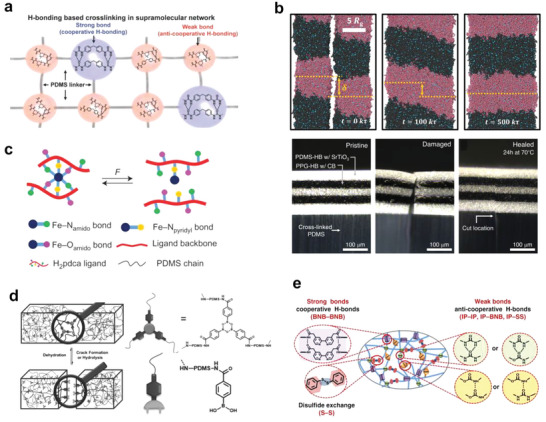
Arrangements of diverse dynamic bonds in PDMS. a–c) Dynamic non‐covalent bonds: a) combinations of strong and weak hydrogen bonds, with the strong transferring elasticity and the weak dissipating energy; reproduced with permission.^[^ ^129^ ^]^ Copyright 2018, Wiley‐VCH. b) multilayered polymeric films undergo autonomous realignment upon damage to minimize interfacial free energy during the healing process, with simulation (top) and microscopic observation (bottom); reproduced with permission.^[^ ^131^ ^]^ Copyright 2023, American Association for the Advancement of Science. c) Fe(III)‐N bonds for modulus enhancement and Fe(III)‐O bonds for energy dissipation, both stabilized by chelation. Reproduced with permission.^[^ ^137^ ^]^ Copyright 2016, Nature Publishing Group. d) Dynamic covalent bonds: reversible equilibrium transition of boroxane bonds in response to humidity stimulation. Reproduced with permission.^[^ ^146^ ^]^ Copyright 2016, Wiley‐VCH. e) Multiple reversible dynamic bonds: the supramolecular polymer network consisting of strong and weak hydrogen bonds and disulfide metathesis. Reproduced with permission.^[^ ^151^ ^]^ Copyright 2020, Nature Publishing Group.

Intrinsic self‐healing can be realized through reversible, dynamic, non‐covalent bonds. Dynamic hydrogen bonding, based on the interaction between a couple of functional moieties (e.g., isofuranone diisocyanate (IPDI),^[^ ^119^, ^120^ ^]^ 4,4′‐methylenebis(phenylisocyanate), Upy,^[^ ^121^ ^]^ and 2‐acrylamido‐2‐methyl‐1‐propanesulfonic acid^[^ ^122^ ^]^) and the PDMS matrix possessing different terminal groups (mainly NH_2_‐PDMS‐NH_2_), has become a widely employed strategy.^[^ ^123^, ^124^, ^125^ ^]^ Wang et al. designed a new hydrogen‐bonded supramolecular elastomer through the condensation reaction of PDMS macromolecules with the diisocyanate coupling agents, and the content of H‐bonds can be precisely adjusted by a rational molecular design, which tunes the flexibility of the polymer chains.^[^ ^126^ ^]^ Besides excellent self‐healing capabilities, other mechanical properties, such as toughness and elongation at break, can also be improved simultaneously. For example, Du et al. introduced a sliding crosslinker (polyrotaxanes) into PDMS and IPDI. As a result, under massive stretching (when the hydrogen bonds had already been broken), the cyclodextrins in the polyrotaxanes slid to dissipate energy, rendering the composite an elongation at break up to 2800% and a breaking strength of 1.05 MPa.^[^ ^127^ ^]^ Liquids such as silicone oil, water, and sweat can further promote the healing effect. The addition of silicone oil to the gelatinized NH_2_‐PDMS‐NH_2_ and IPDI condensates swelled and diluted the polymer chains, which improved the mobility of the urea groups formed on the polymer chains and thus facilitated rapid self‐healing.^[^ ^128^ ^]^ Yao et al. prepared a self‐healing PDMS based on hydrogen bonding between Upy and NH_2_‐PDMS‐NH_2_, and the reversible dissociation/association of the hydrogen bonds was promoted in water. The low permeability of the PDMS phase allowed the water molecules to pass through the polymer network to exchange with UPy motifs, while the hydrophobicity of PDMS localized the UPy‐rich microphases to ensure reversible dissociation and reformation of the hydrogen bonds between UPy motifs. Eventually, 98% of the mechanical properties were recovered in water at 70 °C in as little as 5 min.^[^ ^121^ ^]^ Kang's team prepared a self‐healing material by a condensation reaction between NH_2_‐PDMS‐NH_2_, 4.4′‐methylenebis (isocyanatophenyl ester), and IPDI. The 4,4′‐methylenebis (phenyl urea) unit formed strong hydrogen bonds to transfer elasticity, while the isophorone bisurea unit formed weak hydrogen bonds to dissipate energy (**Figure** **9**a). Due to the synergistic effect, the material exhibited an elongation at break of up to 1200%, and fracture energy of 12 000 J m^−2^, respectively. The mechanical properties of the material can be almost completely restored after 24 h of repair in water or bionic sweat.^[^ ^129^, ^130^ ^]^

Addressing the growing demand for multifunctional, self‐healing devices that can handle increasing complexity, Bao et al. ingeniously integrated bisurea bonds generated by MPU and IU into PDMS and polypropylene glycol (PPG), respectively. Altering the MPU:IU ratio and backbone's average molecular weight facilitated healing kinetics across a broad temperature range (30 – 100 °C). PDMS and PPG have immiscible backbones but share the same dynamic bonds to maintain interlayer adhesion. Interestingly, these multilayered polymeric films underwent autonomous realignment post‐damage to minimize interfacial free energy during the healing process (see Figure 9b). This design concept extends to numerous molecular systems, enabling the production of thin film devices incorporating conductive, dielectric, and magnetic particles, demonstrating 96% self‐healing capability.^[^ ^131^ ^]^

Reversible ionic interactions also play a part in self‐healing PDMS, such as the ionic bonding between amines and acids,^[^ ^132^, ^133^ ^]^ and carboxyl groups and ZnO. The mechanical properties and self‐healing ability of dynamic ionic polymers can be modulated by changing the crosslinking density of the PDMS prepolymer and the molar ratio of –COOH/ZnO.^[^ ^134^ ^]^ Metal ions complex with certain ligands on PDMS terminals or side groups to form coordination bonds, which undergo dissociation and complexation to confer self‐healing properties to PDMS. Bipyridines grafted onto PDMS can form coordination bonds with Zn^2+^ and Fe^2+^ due to their well‐defined coordination geometries.^[^ ^135^, ^136^ ^]^ Bao and co‐workers crosslinked PDMS oligomers containing 2,6‐pyridinedicarboxamide groups with FeCl_3_ to form highly stretchable PDMS elastomers that were able to undergo self‐healing at a remarkably low temperature of −20 °C (Figure 9c).^[^ ^137^ ^]^ To further improve the strength of the elastomer, their team introduced Zn(II)‐carboxylate interactions in PDMS to achieve a combination of stiffness (Young's modulus value of up to 480 MPa) and self‐healing capability.^[^ ^138^ ^]^

Reversible dynamic covalent bonds, such as disulfide bonds,^[^ ^139^, ^140^, ^141^ ^]^ imine bonds,^[^ ^142^, ^143^, ^144^ ^]^ and borate ester/boroxyl bonds,^[^ ^145^, ^146^, ^147^ ^]^ have been explored to construct self‐healing PDMS. Reversible imine bonds are formed through amine‐terminated PDMS or by grafting functional groups containing primary amines to PDMS through Schiff base reactions^[^ ^142^ ^]^ between primary amines and aldehydes or ketones.^[^ ^143^ ^]^ Boronic ester bonds are often obtained through condensation reactions between boronic acids and 1,2‐ or 1,3‐diol compounds.^[^ ^145^ ^]^ Lai et al. prepared a water‐enabled healing material based on a boron‐oxygen covalent bonding system through the amidation of arylboroxanes with NH_2_‐PDMS‐NH_2_. As shown in Figure 9d, in a humid environment, the surface‐exposed boric acid generated a large amount of free boric acid, resulting in the decrosslinking of PDMS chains. This process was reversed after water removal, causing complete healing.^[^ ^146^ ^]^ Ma et al. reported the synthesis of an ultrathin (less than 10 nm‐thick) film with a PDMS network and dynamic boronic acid ester crosslinking that exhibited long‐term hydrophobic properties.^[^ ^147^ ^]^

It is difficult to achieve both satisfactory self‐healing capability and mechanical properties relying on a sole dynamic bond. Including multiple reversible dynamic covalent/non‐covalent bonds in one material holds the promise to break this bottleneck.^[^ ^148^, ^149^ ^]^ Yang et al. synthesized transparent (92% in the visible spectrum) self‐healing PDMS elastomers with dynamic intermolecular hydrogen bonds, reversible imine bonds, and highly flexible Si‐O chains by reacting NH_2_‐PDMS‐NH_2_ and IPDI and a Schiff base reaction with benzaldehyde. The resulting elastomers exhibited a high stretchability value of 1670% and a healing efficiency value of 89% and 78% in water and artificial sweat, respectively.^[^ ^150^ ^]^ In another study, the combination (Figure 9e) of disulfide bonds (S–S), strong hydrogen bonds, and weak hydrogen bonds gave rise to an elongation at break value of 14000% and the encouraging self‐healing speeds in a variety of extreme environments (e.g., ultralow temperature (−40 °C), high concentration brine, and strong acids/bases).^[^ ^151^ ^]^

#### 3.1.7 Dielectric Properties

**Figure 10:**
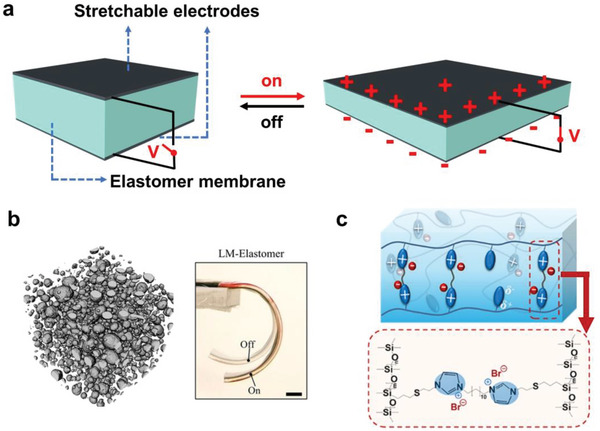
Dielectric property of PDMS composites. a) Structure and operation mode of DEA, where the electrostatic gravitational force between the two electrodes under external voltage causes the elastomer film to be compressed. b) 3D microstructure of LM inside PDMS, showing the homogeneous dispersion of LM droplets of the composite in the PDMS. Image of the DEA of LM‐PDMS elastomeric nanocomposite under voltage for one driving cycle. Reproduced with permission.^[^ ^164^ ^]^ Copyright 2019, Wiley‐VCH. c) PDMS elastomer prepared by cross‐linking reaction between multifunctional imidazole grafted PDMS and bis‐IL. Reproduced with permission.^[^ ^172^ ^]^ Copyright 2022, American Chemical Society.

PDMS is a promising candidate polymer,^[^ ^152^ ^]^ despite its low dielectric constant (ε = 2.5–3.0 at 1 kHz), for the dielectric elastomeric actuator (DEA), which consists of a soft elastomeric film with compliant electrode patterns on both sides, enabling the conversion of electrical energy to mechanical work. As shown in **Figure** **10**a, the application of an external electric field across these electrodes induces an electrostatic attractive force between opposite charges, generating electrostatic stress or pressure on the film. Consequently, the film undergoes a decrease in thickness with an increase in horizontal area.^[^ ^153^ ^]^ PDMS's relatively low modulus as well as physicochemical inertness^[^ ^154^, ^155^ ^]^ result in fast response and low dielectric loss.^[^ ^156^ ^]^ Especially, softer PDMS, either by decreasing crosslinking density or adopting bottlebrush PDMS^[^ ^111^ ^]^ are more suitable for driving DEA at lower voltages.^[^ ^157^ ^]^

DEA's performance can be enhanced by raising the dielectric constant of the DEA film, leading to increased capacitance and less required electric field strength.^[^ ^157^ ^]^ Elastomer composites have traditionally been employed to augment dielectric constant, but this necessitates a balance between high dielectric constant, reduced dielectric loss, and low Young's modulus.^[^ ^158^ ^]^ Nanofiller behavior hinges on their type, quantity, and interaction with the polymer.^[^ ^159^ ^]^ Numerous studies have employed nanofillers like multi‐walled carbon nanotubes (MWCNT)^[^ ^160^ ^]^ and graphene layers^[^ ^161^ ^]^ to modulate polymers' dielectric behavior.^[^ ^162^ ^]^ The nanofiller and PDMS interface affects the dielectric properties of the composites significantly. Noteworthy is Huang et al.’s work, where carbon black particles encapsulated in PDMS embedded in silicone resin significantly enhanced the composite's dielectric constant (by 244%) while preventing carbon black conductivity, yielding high breakdown voltage and reduced dielectric loss. This technique also saw increased bending amplitude and strain in composite DEA.^[^ ^163^ ^]^

To prevent nano‐fillers from decreasing elastic mechanical properties of composite films, Majidi et al. used liquid metal EGaIn as the filler. As shown in Figure 10b, their study showed that high‐concentration nanoscale EGaIn improved the dielectric constant without significant losses in elasticity, tensile properties, or dielectric breakdown strength. Despite minor hardening through incorporating smaller liquid metal, the composites remained soft and highly deformable.^[^ ^164^ ^]^ In addition, high‐dielectric inorganic fillers, such as ZnO,^[^ ^165^ ^]^ TiO_2_,^[^ ^166^, ^167^ ^]^ BaTiO_3_,^[^ ^168^ ^]^ BN,^[^ ^169^ ^]^ and conductive carbon,^[^ ^170^ ^]^ can enhance an elastomer's dielectric constant without altering its dielectric attributes. For example, PDMS‐coated TiO_2_ boosts the breakdown strength of Styrene‐ethylene‐butadiene‐styrene rubber film from 44 to 54.8 V µm^−1^, an increase ideally suited for facilitating high‐strain EDA operation at elevated voltages.^[^ ^171^ ^]^

Another strategy to improve polymer's dielectric property involves chemical modification of the backbone. Skov et al. presented a novel method using a cross‐linking reaction between imidazole‐grafted PDMS and bis(1‐ethylene‐imidazole‐3) brominated ionic liquid (bis‐IL) (see Figure 10c). The resultant IL‐elastomers amplified the dielectric constant by 200% and significantly decreased the modulus (to ≈0.04 MPa), enabling greater strain and lower voltage. Consequently, the developed dielectric actuator demonstrated 20% area strain at 15 V/µm.^[^ ^172^ ^]^ In addition, vinyl‐functionalized Dipole (N‐allyl‐N‐methyl‐p‐nitroaniline) can also be used as a high dielectric grafting molecule in PDMS matrix, increasing the dielectric constant of PDMS from 2.84 to a maximum of 6.15.^[^ ^173^ ^]^

### 3.2 Functionality Emerging at PDMS Surface

#### 3.2.1 Physical and Chemical Modification of PDMS Surface

Surface properties, such as surface energy, morphology, wettability, and adhesion, play a decisive role in various applications. The PDMS surface can be modified by both physical and chemical approaches. The former mainly refers to the construction of micro‐ or nano‐scale periodic structural patterns on the surface, some inspired by nature. The latter includes the precise control of the chemical composition and molecular identity of the surface layer of PDMS through surface treatment and chemical grafting. The surface modification of PDMS is very extensive and is based on the powerful silane chemistry,^[^ ^174^ ^]^ which has been used to make stretchable chemical patterns, gradients, and molecular layers. With the synergistic effect of physical and chemical modifications, novel surface functions can be created and empower numerous practical, real‐life applications.

**Figure 11:**
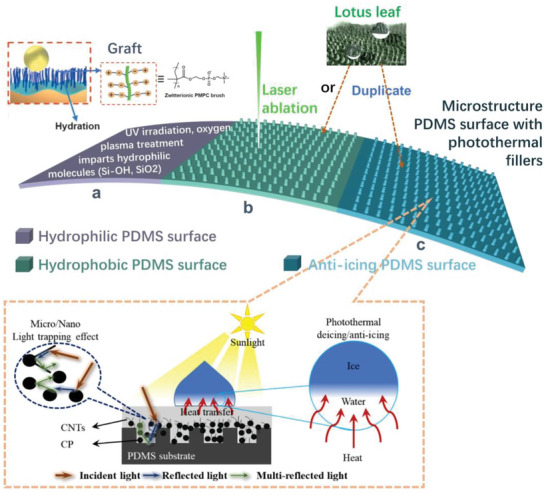
Modifications of the PDMS surface generating diverse properties: a) hydrophilic, b) superhydrophobic, and c) icephobic. a) Multiple methods are used to produce hydrophilic PDMS surfaces. Inset shows the superhydrophilic zwitterionic polymer brush grafted onto the PDMS surface. Reproduced with permission.^[^ ^183^ ^]^ Copyright 2020, Wiley‐VCH. PDMS microstructured arrays depicted in (b) and (c) can be prepared by duplicating the microstructure of the lotus leaf surface or laser etching. c) Illustration of the ray trace and photothermal anti‐icing mechanism in the modified microstructure. Reproduced with permission.^[^ ^220^ ^]^ Copyright 2022, Elsevier.

Vinyl‐terminated PDMS is inherently hydrophobic with a low surface energy (21.3 mJ m^−2^).^[^ ^27^ ^]^ Water droplets form a spherical shape on the PDMS surface, with a WCA of ≈110 °C. Hydrophilic PDMS surfaces (**Figure** **11**) are essential for microfluidic chips and cell culture. For instance, a straightforward surface modification employing amino acid‐functionalized self‐assembled monolayers renders the PDMS surface hydrophilic, thereby furnishing an optimal environment to facilitate cellular maturation.^[^ ^175^ ^]^ Besides UV irradiation, oxygen plasma treatment (see **Table** **5**) can introduce hydrophilic moieties (Si‐OH, SiO_2_) to replace the methyl groups (Si‐CH_3_) on the PDMS surface.^[^ ^176^, ^177^, ^178^, ^179^, ^180^, ^181^ ^]^ A further polyvinyl alcohol layer generated by dip coating extended the hydrophobicity for two months.^[^ ^182^ ^]^ He et al. grafted poly(acryloyloxyethyl phosphorylcholine) (PMPC) onto an Au‐coated PDMS film, which reduced the WCA to 0° and formed a self‐cleaning electrode.^[^ ^183^ ^]^ It was also reported that surface modification of PDMS foam with polyacrylic acid could maintain superhydrophilicity (≈1° of WCA) for up to 18 months.^[^ ^184^ ^]^

**Table 5:** Functional PDMS surfaces with hydrophilic or superhydrophobic properties.

Modification approach	WCA (°)	Slide angle (°)	Featured application	Reported year
Low‐energy electron beam irradiation	60 in 1 min		Cell‐adherent	2018^[^ ^178^ ^]^
Loading of polyvinyl alcohol and plasma treatment	7 immediately after plasma oxidation		Drug delivery	2017^[^ ^179^ ^]^
PMPC brush on Au‐coated PDMS surface	10		Self‐cleaning skin electrode	2020^[^ ^183^ ^]^
Polyacrylic acid coating	9		Portable pressure pump	2019^[^ ^184^ ^]^
Zn coating	2		Water collector	2021^[^ ^180^ ^]^
Pt deposition on silica PDMS aerogel surface	0		Water harvesting	2017^[^ ^181^ ^]^
Hierarchical micro/nano structures	146 (159 after flame treatment)		Sweat‐resistant wearable TENG	2022^[^ ^190^ ^]^
Hexagonal convex microlense array	156	4	Water collector	2021^[^ ^180^ ^]^
Microstructure from solvent‐induced polycarbonate mold	172	< 1	Self‐cleaning surface	2020^[^ ^27^ ^]^
CO_2_ laser engraver irradiation	155	5	Superhydrophobic and wearable strain sensors	2022^[^ ^187^ ^]^
CO_2_ laser engraver irradiation	153		Light‐driven floating device	2017^[^ ^191^ ^]^
Labyrinth‐like wrinkles of Zn film	169	0	Self‐cleaning surface	2022^[^ ^195^ ^]^
ZnSn(OH)_6_ layer coating	158	1	Self‐cleaning coating	2017^[^ ^196^ ^]^
Coating of fluorinated nanoparticle/binder	157	<1	Transparent self‐cleaning surface	2017^[^ ^359^ ^]^
With microstructure of lotus leaf surface	>170	2	Anti‐icing	2016^[^ ^218^ ^]^
With cauliflower‐like micro‐nano structure	161	1	Anti‐icing	2022^[^ ^221^ ^]^
With micro‐nano‐NW triple structure	159	2	Anti‐icing	2022^[^ ^360^ ^]^
Candle soot coating	162		Bimorph actuators	2021^[^ ^296^ ^]^
Microstructure and carnauba wax	169		Detection of adulterant rhodamine B	2022^[^ ^361^ ^]^

Superhydrophobic surfaces (Figure 11), usually characterized by a WCA > 150°,^[^ ^185^, ^186^, ^187^ ^]^ have attracted much attention due to their potential applications in water‐proofing, self‐cleaning, and anti‐icing devices.^[^ ^31^, ^188^ ^]^ Constructing micro‐ or nanostructures on the PDMS surface via pre‐fabricated templates or laser writing is a general approach to enhance WCA,^[^ ^189^ ^]^ since these techniques allow a layer of air to be inserted between the PDMS surface and water droplets.^[^ ^17^ ^]^ Li et al. replicated the lotus leaf surface micro‐nano structures on PDMS and achieved a WCA of 146°. Interestingly, subsequent flame treatment, which caused the formation and shedding of SiO_2_ to produce a complex architecture at the tip of the microstructure, further enhanced the WCA to 159.4°.^[^ ^190^ ^]^ Sun et al. prepared novel, light‐driven floating devices with superhydrophobic surfaces (WCA = 153°) by laser engraving asymmetric structures on PDMS.^[^ ^191^ ^]^ Thanks to the resulting light‐absorbing and superhydrophobic carbonized surface layer, these devices could be driven by various light sources to produce swift linear or rotational motions with superb photothermal conversion efficiency.

Another approach to improve the hydrophobicity is to reduce the surface energy of PDMS via surface chemical modification. Fluorochemicals have been widely used for this purpose because of the fluorine atom's strong polarity and low surface tension. Furthermore, when elastomer mechanics and surface chemistry are coupled, surfaces featuring mechanodynamic molecular layers, i.e., mechanically assembled monolayers (MAMs), can be achieved. The impact of these surface modification approaches on the surface micromorphology of PDMS has been investigated.^[^ ^192^ ^]^ In 2000, Jan Genzer pioneered the fabrication of MAMs by using semi‐fluorinated (SF) molecules on ultraviolet ozone‐treated (UVO‐treated) PDMS surfaces. They achieved tunable graft densities and chain stacking by combining self‐assembly of surface graft molecules with mechanical manipulation of graft sites on PDMS surfaces. This MAM formation results in a superhydrophobic surface possessing outstanding and enduring barrier properties. Remarkably, the superhydrophobic characteristics remain unaltered even after extended exposure to water, which would typically induce the reconstruction of conventional SF self‐assembled monolayers.^[^ ^193^ ^]^ Similarly, fluorinated (1H,1H,2H,2H‐perfluorodecyltriethoxysilane) silica particles were blended with a PDMS two‐component system and sprayed on the surface of PDMS films with microstructures. Upon curing, the surfaces demonstrated enhanced hydrophobicity and robust corrosion resistance, with a WCA of 153.2° and slide angles of merely 3°.^[^ ^194^ ^]^ Recently, environmentally friendly modification reagents alternative to fluorochemicals have been developed, such as ZnSn(OH)_6_ and Zn layers.^[^ ^195^, ^196^ ^]^

Mechanically reversible surfaces have been used to modulate surface wettability, and surfaces based on elastomeric materials with mechanically controlled wrinkles and microstructures can provide simple and fast water repellency.^[^ ^197^ ^]^ However, surface microcracking and complex fabrication procedures limit the scope of application of these materials.^[^ ^198^ ^]^ To address this, Mazaltarim et al. successfully fabricated layered structured surfaces supported by PDMS elastomers, comprising nano‐porous films with mechanically adjustable microcrack networks. The UVO‐oxidized PDMS surface forms a heterogeneous oxide gradient which, under uniaxial mechanical strain, yields parallel microcrack networks with controlled anisotropy. After chemical etching, the uppermost hydrophilic/highly oxidized silica layer is eventually removed, exposing the less oxidized/more hydrophobic silica. This culminates in the formation of layered micro/nanostructured superhydrophobic surfaces.^[^ ^175^ ^]^ In addition, the PDMS surface can be deliberately transformed from hydrophilic to hydrophobic in a facile manner. The same group found that through repeated film stretching, the hydrophobicity was recovered from the hydrophilic surface of oxygen plasma‐treated PDMS, mainly due to the cracking of the pre‐formed surface SiO_x_ layer and the migration of uncured monomers from the bulk matrix to the surface. The WCA of the material (ranging from 5° to 90°) can be adjusted by controlling the stretching amplitude and cycle number.^[^ ^199^ ^]^

**Figure 12:**
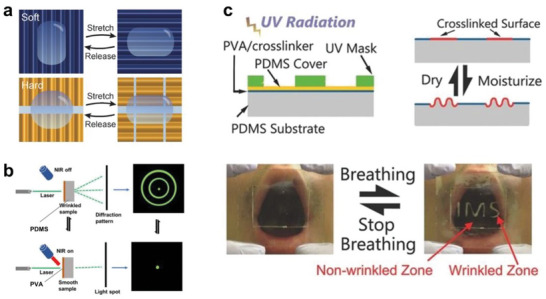
Functional wrinkles on a PDMS surface. a) Switchable anisotropic wrinkles generated by stretch and release of the polymer. Reproduced with permission.^[^ ^207^ ^]^ Copyright 2017, Wiley‐VCH. b) Reversible wrinkles controlled by NIR irradiation for dynamic light grating. Reproduced with permission.^[^ ^209^ ^]^ Copyright 2018, American Association for the Advancement of Science. c) Reversible wrinkles of PVA are regulated by moisture, which affects the reflection of the film. Reproduced with permission.^[^ ^210^ ^]^ Copyright 2017, Wiley‐VCH.

Surface wrinkling is a simple and novel method for fabricating micropatterns by imparting unique and fascinating physical, chemical, and biological properties.^[^ ^200^ ^]^ The formation of controllable wrinkles on elastomeric surfaces such as PDMS is essential for the development of technologies such as microfluidics, water harvesting, energy generation, bioanalysi,s and microreactor design. Chaudhury and Whitesides pioneered demonstrating how chemical wettability gradients could facilitate liquid microdroplet transportation on surfaces.^[^ ^201^ ^]^ The generation of surface micromorphology (wrinkles) caused by mechanical mismatch between the hard surface layer and the soft underlying polymer has been extensively investigated.^[^ ^192^, ^202^, ^203^, ^204^ ^]^ Mazaltarim et al. further explored this concept using mechanically‐induced micro‐wrinkles on pre‐strained, UVO‐treated PDMS films. The resulting surface silica layer reacted to strain release by forming controlled micro‐wrinkles. Following a brief exposure to oxygen plasma to enhance surface Si‐OH concentration, they manipulated mechanical strain to dynamically dictate wrinkled microtextures' spacing/amplitude, hence, governing microdroplet transport. The velocity, displacement, and critical radius of microdrop transport can be tuned, and this work demonstrates a new way to design smart materials with programmable transport properties.^[^ ^205^ ^]^ In addition, their team has investigated the effect of anisotropic PDMS surface roughness on droplet spontaneous transport.^[^ ^206^ ^]^ In particular, the correlation between chemical gradient strength, micro‐wrinkle orientation, and droplet velocity/trajectory was investigated. Knowing the details of this process shows the potential to unlock new fluid handling capabilities, which are essential for the development of next‐generation intelligent surfaces. Recently, researchers found that the wrinkle direction can be manipulated by controlling the stretching scale. Soft fluoropolymer was deposited on a pre‐strained PDMS film via reactive ion etching, and the crack‐free surface wrinkling was generated perpendicularly to the pre‐stretch direction after release. Then, the bilayer film was stretched again along the pre‐stretch direction. It was observed that the existing wrinkles gradually disappeared and new wrinkles perpendicular to the original ones formed under strain exceeding the preload strain (**Figure** **12**a). The wrinkled surface is promising for switchable anisotropic liquid spreading.^[^ ^207^ ^]^

Reversible modulation of wrinkles can be realized with stimuli such as temperature and moisture. Wrinkles were formed on a PDMS‐graphene bilayer structure after annealing at 120 °C due to the asymmetric CTE of the two layers and were utilized for ultrasensitive piezoresistive sensing.^[^ ^208^ ^]^ Similarly, Li et al. generated reversible wrinkle patterns upon heating and cooling, with a polymer (such as polysulfone or polyacrylonitrile) layer on the CNT‐PDMS film. The CNT‐PDMS layer expanded under near‐infrared (NIR) irradiation due to the photothermal effect of CNT, leading to the reversible disappearance of the wrinkles (Figure 12b). This work sets the first example of dynamic NIR grating and is promising for application in electronic devices such as dynamic optical gratings and displays.^[^ ^209^ ^]^ Furthermore, inspired by the reversible modulation of wrinkles on human skin (e.g., fingers) at different moisture levels, Zeng et al. designed a moisture‐responsive system with a hydrophilic PVA film adhered to a hydrophobic PDMS layer. As shown in Figure 12c, the PVA layer swelled upon exposure to elevated humidity levels, leading to the generation of wrinkles and the reduction in optical transmittance.^[^ ^210^ ^]^

Icing is a common natural phenomenon affecting human activities and can cause severe safety problems to aircraft, power grids, transmission lines, roads, and other devices and infrastructure.^[^ ^211^ ^]^ To achieve an icephobic surface, the ice adhesion strength (*τ*_ice_ = *F* (adhesive force) / *A* (attachment area)) should be kept at less than 100 kPa.^[^ ^212^ ^]^ With its low surface energy, PDMS was the material of choice in early attempts to reduce ice adhesion.^[^ ^213^ ^]^ Recently, it has been found that conventional PDMS can be further optimized to provide anti‐icing activity. On one hand, hydrophobic PDMS surfaces can increase the ice crystal nucleation potential to prolong the nucleation time or weaken the adhesion. On the other hand, PDMS surfaces with low elastic modulus are prone to microscale deformation, which reduces the shear ice adhesion. For example, the elasticity and surface wettability of PDMS can be adjusted to modify ice adhesion by simply mixing different ratios of the prepolymer with a curing agent^[^ ^214^ ^]^ or silica nanoparticles.^[^ ^215^ ^]^ It was found that a 3:1 mass ratio of PDMS prepolymer to PDMS curing agent renders both low modulus and high surface hydrophobicity, resulting in ice bonding strength below 10 kPa, which is the ultralow limit of ice adhesion. Furthermore, Golovin et al. found that when the interface width of the ice‐attached surface is certain, the ice adhesion force approaches a limit as the length of the ice‐attached interface increases. In this scenario, the interfacial toughness controls the adhesion of the ice layer (the lower the toughness, the easier for the ice to fall off). Based on this theory, a PDMS coating layer with reduced thickness (1‐2 mm) and enhanced plasticity (medium‐chain triglyceride oil) were produced, with a low interfacial toughness of 0.12 J m^−2^ and a *τ*_ice_ of less than 4 kPa. Impressively, 1 cm‐thick ice on a 1 m^2^ aluminum plate coated with PDMS will fall off naturally by gravity alone.^[^ ^216^ ^]^

Rough surfaces with micro‐ or nanostructures further improve ice‐repelling performance.^[^ ^217^ ^]^ Wang et al. grew ZnO NWs (≈30 nm in diameter) on the surface of PDMS films with a lotus leaf surface structure to form a hierarchical micro‐/nanomorphology and then treated the surface with heptadecafluorodecyl tri‐propoxy silane (FAS‐17) to reduce its surface energy. Due to the repellent effect of ZnO NWs on condensation droplets and the suspension effect of PDMS microstructure on the agglomeration of tiny condensation droplets, this structured surface can maintain water and ice resistance for three months at −20 °C and 90% humidity.^[^ ^218^ ^]^

Besides these well‐developed, passive anti‐icing strategies, recent research has focused on the active de‐icing approaches, usually by introducing photothermal fillers (such as CNTs, carbon powders, and candle soot), that can convert environmental light to heat. Chen et al. loaded poly(cyclotriphosphazene‐co‐4,4′‐sulfonyldiphenol) Fe_3_O_4_ onto acidified CNTs and then mixed it with PDMS to form microarray structures on the composite surface by compression molding and magnetic attraction. When a magnetic field was applied during curing, the magnetic Fe_3_O_4_ particles dragged the CNT filler toward the surface layer, which ultimately shortened the heat transfer path of the filler during the photothermal process. The magnetic particles were responsible for improving the warming efficiency, which was increased by about 46% under NIR irradiation, and frozen droplets melted within 20 s.^[^ ^219^ ^]^ The well‐designed complex surface structure can direct the light path to propagate more randomly to improve the absorbance and promote active de‐icing efficiently by warming the surface rapidly. Xie et al. sprayed a layer comprising a mixture of carbon powder and CNTs onto the surface of PDMS microcolumn arrays and achieved a low freezing temperature (≈25 °C) and long icing delay time (≈13 min). The photothermal conversion efficiency of the carbon powder and CNTs was as high as 61% due to the light reflection induced by the microcolumn array structure. Consequently, the ice melted in only 300 s under sunlight and at room temperature.^[^ ^220^ ^]^ Chen et al. grew cauliflower‐like structures of zinc and antimonated zinc on the surface of aluminum microcones and spin‐coated a thin PDMS layer. The surface of the obtained hierarchical structure exhibited a “light trapping effect” with light absorption up to 97.3%. The transparent PDMS layer, with its low thermal conductivity, ensured that the heat generated by light‐induced damped oscillations of the free electrons was not quickly dissipated, and the surface temperature rose to about 89 °C at room temperature with 2 sun illumination for 300 s.^[^ ^221^ ^]^ Black candle soot is characterized as an excellent photothermal material.^[^ ^222^, ^223^ ^]^ Wu et al. deposited a layer of candle soot particles (30–40 nm in diameter) on glass and assembled the component layer structure from nanoscale carbon. The stability of the bare soot coating was enhanced by the chemical vapor deposition of a thin SiO_2_ shell over the candle soot. Then, under UV light, the hydroxyl groups emerged on the surface of SiO_2_, and the siloxane bond of PDMS was partially cleaved to form a covalent bond with the silicon group through the Si–O–Si bond, thus grafting the PDMS brush. The hierarchical surface captured sunlight by multiple internal reflections with only < 1% of sunlight reflected or scattered, which allowed the surface temperature to increase by 53 °C under 1 sun irradiation. No ice could form even at −50 °C, and the super‐hydrophobic surface (WCA = 163°) enabled the melted water to immediately slide away, leaving a clean and dry surface.^[^ ^224^ ^]^

**Figure 13:**
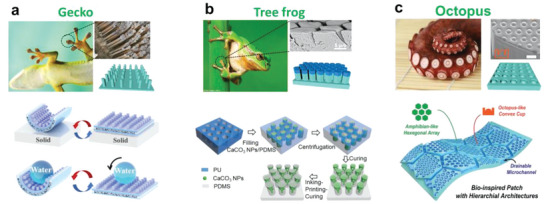
PDMS‐based adhesives inspired by various organisms in nature: a) gecko toe, b) tree frog toe, and c) octopus sucker. Detailed microstructure and chemical innovations are shown below: a) When LMF/PDMS is in contact with a solid, low adhesion strength can be obtained in the curved state (low contact area), while high adhesion strength can be obtained in the horizontal state (high contact area). Similarly, reversible liquid adhesion can be achieved by controlling the contact state between water droplets and the upper surface of LMF/PDMS. Reproduced with permission.^[^ ^236^ ^]^ Copyright 2023, Springer Nature. b) PDMS microcolumn arrays with modulus gradients; Reproduced with permission.^[^ ^241^ ^]^ Copyright 2021, Wiley‐VCH. c) Schematic diagram of the hierarchical structure of the microchannel network of the treefrog toe and the raised circular holes of the octopus’ sucker. Reproduced with permission.^[^ ^244^ ^]^ Copyright 2019, Wiley‐VCH.

PDMS‐based materials are popular in the field of bionic adhesion because of their chemical flexibility, relatively low modulus, and ease of processing,^[^ ^31^ ^]^ and can even outperform their biological counterparts in medical, bioelectronic, biomechanical and robotic applications. The gecko is an intriguing animal known for its ability to climb rapidly up smooth vertical surfaces due to the hierarchical micro‐ and nanostructures on the bottom surfaces of its feet that creates strong adhesion to the wall by accumulating van der Waals forces (**Figure** **13**a).^[^ ^225^, ^226^ ^]^ This spurred the development of a skin‐attachable strain sensor using an adhesive PDMS film with a micropillar structure and a spatula tip.^[^ ^227^ ^]^ Furthermore, inspired by the bending capability of the flexible foundation element of the gecko's lamellar skin, Hu et al. prepared a discretely supported dry adhesive film consisting of a micropillar array surface and a bendable film base that could adhere to rough surfaces.^[^ ^228^ ^]^ Enhancing the adhesion strength requires uniform load distribution across the contact interface.^[^ ^229^ ^]^ Song et al. prepared an adhesion‐based soft gripping system consisting of a PDMS micropillar array surface with a pressure‐controlled deformable PDMS body.^[^ ^226^ ^]^ The system forced more microcolumns to come in contact with the adherent surface under external pressure, resulting in uniform load sharing even on 3D object surfaces (rough and curved surfaces), and a 14‐fold improvement for adhesive force was observed.

Scientists also developed materials to mimic gecko's ability to detach its toes from a surface.^[^ ^230^ ^]^ The conventional detachment strategy is through the photothermal effect via adopting light absorbers, such as QDs^[^ ^231^ ^]^ and graphene.^[^ ^232^ ^]^ Recently, Zhao et al. reported magnetically driven adhesives by introducing magnetic NdFeB particles in mushroom‐shaped adhesive heads. The sample produced an adhesive force of 4.32 mN toward the substrate under pre‐pressure. However, when a weak magnetic field (97 mT) perpendicular to the surface was applied, the adhesive head bent outward immediately (less than 0.5 s), which resulted in a gap between the adhesive head and the substrate, leading to a substantial reduction in the adhesive force (0.25 mN). The adhesive force could be precisely regulated by nesting the array of adhesive heads according to the weight of the target object and the adhesion area.^[^ ^233^ ^]^

Exploration into the effects of harsh space environments on adhesive properties remains limited. Notably, PDMS elastomers' mechanical properties hinge on temperatures, leading to significant adhesive property fluctuations in varying temperatures, and especially, the performance decay in cold environment. For this reason, Li et al. introduced phenyl as a side group to the PDMS backbone via anionic ring‐opening polymerization, and the PDMS elastomer showed amorphous properties due to the reduced regularity of the macromolecular chain. The elasticity could be better maintained over a wider temperature range than that of the original PDMS. The product microfibrillar adhesives maintained optimal adhesion even below −120 °C.^[^ ^234^ ^]^

In addition to dry adhesion, underwater adhesion control can be achieved by modifying the microcolumn array surface. For example, by grafting thermally responsive poly‐N‐isopropylacrylamide (PNIPAM) containing molecules on the surface of PDMS micropillar arrays, switchable liquid adhesion was achieved due to the different hydrophilicity of PNIPAM in different temperature ranges.^[^ ^235^ ^]^ Moreover, shape changes caused by bending, stretching or compression can be achieved for liquid adhesion control. Liu et al. prepared a liquid metal foam/PDMS (LMF/PDMS) composite, as shown in Figure 13a, in which the central part consists of PDMS‐filled LMF with one side coated by a smooth layer of PDMS (with tunable solid adhesion) and the other side by a PDMS layer with surface micropillars (with controlled liquid adhesion). Due to the reversible change between the solidified and melted states of LM and the excellent elasticity of PDMS, the composite film exhibited excellent shape memory effects. During the metamorphic process, the bilateral solid and liquid adhesion forces could be adjusted in a wide range (with solid adhesion strength 0.8–43.9 kPa, liquid adhesion 24.3–17.1 µN) due to the controllable contact area.^[^ ^236^ ^]^

The research on biomimic adhesion systems has moved well beyond geckos. Treefrogs (Figure 13b) exhibit strong adhesion through mechanical interlocking on a variety of surfaces,^[^ ^237^ ^]^ and octopus (Figure 13c) suckers maintain fixation with the substrate through the gaps created by the collapse of their structure.^[^ ^238^ ^]^ Inspired by the nano‐concave tops of epidermal cells on the toe pads of tree frogs, Xue et al. designed an array of composite micropillars with nanopits on the surface. They embedded PS nanoparticles inside PDMS micropillars and formed nanopits after etching, and achieved greater wet adhesion. Most of the liquid between the array and the corresponding surface is extruded under a certain loading force, so the liquid remaining in the nanopits forms multiple nanoscale liquid bridges within the contact area of a single micropillar and introduces a suction effect at the interface. Adhesion resulted from multiple liquid bridges, suction effects, and direct solids contacts, exemplifying 36.5 times the forest of tree frog toes.^[^ ^239^ ^]^ Furthermore, combining PDMS micropillar arrays with vinyl‐modified rigid PS nanopillars managed to alter stress distribution, enhancing adhesion and friction.^[^ ^240^ ^]^ In addition, Liu et al. distributed CaCO_3_ nanoparticles in PDMS microcolumns with a concentration gradient by centrifugation (Figure 13b), and found that the adhesion force reached its maximum (13 mN) when the modulus gradient rate was 403.4 kPa µm^−1^, due to the fact that this type of gradient can build compliant contacts with the uneven and misaligned surfaces and possesses high wear resistance.^[^ ^241^ ^]^

Octopus‐inspired micropatterned surfaces offer rough and wet skin adhesion. For instance, PDMS‐based conductive polymer composite films were prepared containing circular pores with dome‐like protruding structures. Under external pre‐pressure, the cavities were separated by contact between the dome‐like structure and the nearby sidewalls, and capillary forces drained the liquid into the internal chamber, thus creating adhesion between the liquid molecules.^[^ ^242^ ^]^ The thermoresponsive hydrogel pNIPAM helps to reduce the intraluminal pressure at high temperatures (>40 °C), resulting in a greater adhesion force.^[^ ^243^ ^]^ Inspired by the microchannel network in the toe pads of tree frogs and the raised circular holes in octopus suction cups, Pang et al. built highly breathable, drainable, and reusable skin patches with a layered structure (Figure 13c). They obtained hexagonal PDMS micropillar arrays with octopus‐like convex cups on the surface by replica molding. The cohesive force causes liquid molecules to flow into the lumen of the suction cups, thus inducing a capillary‐assisted suction effect that enhances their adhesion strength on moist substrates.^[^ ^244^ ^]^

A TENG is an emerging technological device that converts mechanical energy into electricity due to the electrostatic charge created by two separate material surfaces through physical contact.^[^ ^245^ ^]^ PDMS, imbued with mechanical stability and hydrophobicity, is often used as a negative electrode for TENG due to its tendency to gain electrons. Cho et al. found that PDMS can accumulate negative charges under the continuous impact friction of raindrops. Importantly, this TENG maintained the high light transmission of PDMS and can be integrated into solar cells without hampering their light‐harvesting capability, resulting in a maximum power density of 1.17 W m^−2^.^[^ ^246^ ^]^

The difference in electron affinity between the PDMS negative layer and the positive counterpart should be maximized to facilitate electron transfer.^[^ ^247^ ^]^ Fluorine‐containing polymers are often used to modify PDMS because F exhibits a higher electron affinity. For example, Li et al. directly mixed PDMS with perfluorododecyl trichlorosilane to prepare a negative electrode that attracted more electrons and increased the dielectric constant, ultimately achieving a high transfer charge of 80 nC. Owing to the superhydrophilicity of PDMS, a voltage of 105.8 V was obtained even under ambient conditions of up to 60% humidity.^[^ ^248^ ^]^ Yang et al. used 1H,1H‐perfluorooctylamine (F_15_‐NH_2_) as a surface modification layer, and the protonated amine groups on F_15_‐NH_2_ were immobilized on PDMS by electrostatic interactions. The highly electronegative perfluoroalkyl chains accumulated at the surface induced favorable surface dipoles for efficient electron transfer between the electrode and the dielectric layer.^[^ ^249^ ^]^ In TENG, a high contact area generates more frictional charges while a higher surface area accumulates more charges.^[^ ^250^ ^]^ A widely employed method is to create a rough surface by laser etching.^[^ ^251^ ^]^ Lee et al. also prepared a PDMS micropillar structure covered by a PDMS layer on both sides, and concluded that the second PDMS layer ensured an increased contact area and a five‐fold improvement in charge accumulation.^[^ ^252^ ^]^

Surface and interfacial bonding technology^[^ ^253^ ^]^ matters in PDMS device applications. PDMS‐based conductive composites are often utilized in electronic devices due to their printability, stretchability, and high electrical conductivity.^[^ ^254^ ^]^ Yet, their low surface energy and limited reactive functional groups lead to weak bonding with other devices, adversely impacting the functionality of stretchable devices.^[^ ^255^ ^]^ PDMS also serves as the fundament in microfluidics,^[^ ^256^, ^257^, ^258^, ^259^, ^260^, ^261^, ^262^, ^263^, ^264^ ^]^where microchannels can form a chip bonded to solid or flexible substrates, such as polyethylene terephthalate,^[^ ^265^ ^]^ polyimide,^[^ ^266^ ^]^ PMMA,^[^ ^267^ ^]^ polyvinyl chloride,^[^ ^268^ ^]^ and polycarbonate,^[^ ^269^ ^]^ via high strength, quality bonding. In addition, PDMS can act as a substrate for enclosing PDMS microchannels,^[^ ^270^ ^]^ with surface activation achieved via oxygen plasma^[^ ^271^ ^]^ and corona treatment processes. This activation replaces PDMS' terminal methyl (‐CH_3_) with a silanol group, enabling the formation of a covalent siloxane bond (Si‐O‐Si) with another activated surface. The surface treatments induce interactions and oxidation, increasing PDMS surface's hydroxyl concentration and intermolecular bonds.^[^ ^272^ ^]^ Alternatively, UV/ozone treatment enables deeper surface modification while ensuring minimum surface damage.^[^ ^15^, ^273^, ^274^ ^]^ Chemical gluing also enables PDMS bonding to materials like thermoplastics through a molecular monolayer with a specific terminal functional group.^[^ ^275^ ^]^ For example, the silanol group of PDMS can be used directly to form bonds with the amino group (─NH_2_) of 3‐aminopropyl‐triethoxysilane^[^ ^267^ ^]^ or the thiol group (─SH) of MPTMS (3‐mercaptopropyl‐trimethoxysilane) functionalized polymers.^[^ ^276^ ^]^

Another challenge related to the PDMS surface is protein adsorption in tissue engineering microfluidics. Zhang et al. successfully mitigated this by covalently forming a slippery coating on PDMS surfaces using acid‐catalyzed hydrolysis and condensation of dimethyldimethoxysilane, which effectively reduced protein uptake.^[^ ^277^ ^]^ Additionally, enhancing PDMS surface energy by adding different quantities of PDMS‐(60–70% ethylene oxide) block copolymers can significantly control fluid flow properties.^[^ ^278^ ^]^ While PDMS microfluidics techniques are relatively mature, for further details, we direct interested readers to the review by Zhao et al.^[^ ^262^ ^]^

#### 3.2.2 PDMS‐Based Bimorph Structure

**Figure 14:**
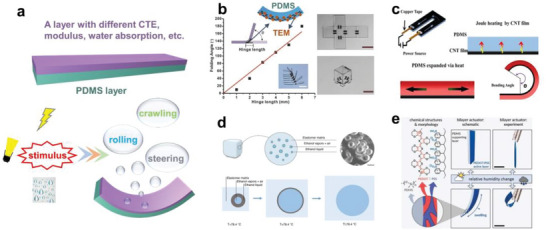
Bimorph actuator based on a PDMS film and an adjacent functional layer. a) Central idea of the stimuli‐responsive bimorph actuator with designed responses to various categories of stimuli and a handful of mechanical action modes. b) Photothermal actuator with TEM as the super‐thermal‐expansive layer. Reproduced with permission.^[^ ^288^ ^]^ Copyright 2017, Wiley‐VCH. Electrothermal actuator with c) CNT as the conductive layer (Reproduced with permission.^[^ ^293^ ^]^ Copyright 2021, Springer Nature) or d) Ni wire as the conductive connector and ethanol bubbles as the super‐thermal‐expansive component (Reproduced with permission.^[^ ^294^ ^]^ Copyright 2017, Nature Publishing Group). e) Humidity‐triggered actuator with PEDOT:PSS as the water adsorption layer. The scale bar represents 1 mm. Reproduced with permission.^[^ ^296^ ^]^ Copyright 2021, Wiley‐VCH.

The bilayer actuator, a stimuli‐responsive soft actuator that combines an active layer and a supporting/passive counterpart, is a type of emerging intelligent structure (**Figure** **14**a). It can be endowed with various movement patterns, including linear, rolling, and steering movements under different stimuli such as an electric field,^[^ ^279^ ^]^ humidity,^[^ ^280^ ^]^ temperature,^[^ ^281^ ^]^ and light.^[^ ^282^ ^]^ Bilayer actuators show great potential for applications in fields such as bionic actuation,^[^ ^12^, ^283^ ^]^ artificial muscles,^[^ ^284^, ^285^ ^]^ and even textiles.^[^ ^286^ ^]^ PDMS bonds tightly with a wide range of materials. In addition, a few key properties introduced in Section 2, such as optical transparency, surface hydrophobicity, high CTE, high elasticity, high compatibility, and low Young's modulus, provide PDMS unique advantages for their application in soft actuating systems.

The general principle for a bilayer actuator is that the distinct physical properties (such as CTE) of the two layers lead to asymmetric expansion when a stimulus such as heat is applied by optical illumination, Joule heating, or direct heat transfer, which eventually leads to bending toward the specific side with the lower CTE (**Table** **6**). The bilayer photo‐responsive actuator generally consists of a PDMS supporting layer and another photothermal conversion layer. Due to the high CTE of PDMS,^[^ ^287^ ^]^ the actuator usually bends toward the photothermal conversion layer. However, there are also exceptions. Tang et al. adopted an active layer of reduced graphene oxide (rGO) and thermally expanded microspheres (TEMs) with ultrahigh CTE (≈2 × 10^−3^ K^−1^) uniformly distributed in PDMS,^[^ ^288^ ^]^ and determined that IR irradiation led to the large volume expansion of TEM, eventually causing the bilayer to bend toward the PDMS side (Figure 14b). Based on the hydrophobicity of PDMS, Wang et al. fabricated a whale‐like robot consisting of a graphene oxide (GO) layer and a PDMS layer operating perpendicular to the water surface, which could mimic the motion of a fishtail under IR irradiation.^[^ ^289^ ^]^ In addition, the porous PDMS layer can be used as an important component in the photothermal active layer, and by depositing graphene on the surface of the pores, a fast bending response (bending up to 110° in 1 s) and a high speed at the water surface of up to 28 mm s^−1^ were demonstrated.^[^ ^290^ ^]^ Xiao et al. directly incorporated an optical fiber taper into a PDMS/gold nanorod hybrid film (photothermal conversion layer) as an internal light source, which enabled high local stimuli intensity and precise control of the deformation sites. This system can be used to handle and move objects of different shapes.^[^ ^291^ ^]^

**Table 6:** Typical PDMS‐based bimorph actuators.

Type of stimuli	Passive layer	Active layer	Bending angle or curvature (time)	Application	Reported year
Photothermal (IR)	PDMS	RGO‐TEM‐PDMS	180° in 30 s	Actuating hinge	2017^[^ ^288^ ^]^
Photothermal	GO‐PDA‐Au	PDMS	60° in 2 s	Crawling robot	2020^[^ ^287^ ^]^
Photothermal (IR)	Graphene‐PDMS	PDMS		Soft robots	2019^[^ ^289^ ^]^
Photothermal (IR)	Porous PDMS‐GO	PDMS	110° in 1.3 s	Soft robots	2022^[^ ^290^ ^]^
Photothermal	GO	PDMS/Au nanorods	270° (180° in 2 s)	Machine gripper	2022^[^ ^291^ ^]^
Joule heating	CNT film	PDMS	324° in 12 s	Machine gripper	2021^[^ ^293^ ^]^
Joule heating	PDMS	Ethanol inside PDMS		Artificial muscle	2017^[^ ^294^ ^]^
Humidity (RH=90%)	PDMS	PEDOT:PSS	1.2 mm^−11^		2021^[^ ^8^ ^]^
Humidity	Candle Soot	PDMS	270° in 2 s	Drivers	2021^[^ ^296^ ^]^
Joule heating and photothermal	CNT	PDMS	235° in 5 s/215° in 1 s	Jumping robot	2017^[^ ^362^ ^]^
Photothermal and humidity	GO/CNT‐PDMS	CNT‐PDMS/GO	90° in 3 s /137°	Crawling robot	2018^[^ ^363^ ^]^
Alkanes and humidity(RH=90%)	GO/PDMS	PDMS/GO	10 cm^−11^	Valve switch	2019^[^ ^298^ ^]^

An electrothermal actuator (ETA) is an intelligent device that converts electricity into mechanical energy by Joule heating.^[^ ^292^ ^]^ Aouraghe et al. adopted a CNT film with high electrical (≈10^5^ S cm^−1^) and thermal conductivity (≈80 W m^−1^ K^−1^) as the electrothermal conversion layer before cutting the PDMS‐CNT bilayer into a U‐shape to achieve uniform circular bending motion around the horizontal axis of the actuator (Figure 14c). At a low drive voltage of 8 V, the composite ETA bent at an angle of 324° in 12 s.^[^ ^293^ ^]^ Miriyev et al. hand‐mixed ethanol with PDMS prepolymer, and the ethanol droplets encapsulated in the PDMS matrix expanded significantly under the resistive heating provided by the nickel‐chromium wire inside PDMS, as illustrated in Figure 14d. The volume expanded up to 915% at 90 °C and could support a gravity force 6000 times greater than itself. When laminated to an inert silicone elastomer layer, the asymmetric stress made it suitable for mimicking natural muscle flexion behavior.^[^ ^294^ ^]^

The mechanical response to changes in humidity is a common phenomenon in nature. For example, a severed leaf shrinks under dry conditions but regains its shape and properties when placed in water.^[^ ^295^ ^]^ The bilayer film actuators inspired by this phenomenon generally comprise an active layer with a more significant response to humidity and a hydrophobic PDMS inert layer that is nearly unreactive. Dingler et al. designed a poly(3,4‐ethylenedioxythiophene): poly(styrene sulfonate)‐PDMS bilayer actuator, which bent toward the PDMS side with a curvature of 1.2 mm^−1^ (Figure 14e) when the relative humidity (RH) was increased to 90%.^[^ ^8^ ^]^ The bending magnitude can be maximized by increasing the hydrophobicity of the inert layer and the hydrophilicity of the active layer.^[^ ^296^ ^]^

Multiple types of stimuli can make contributions synergistically by clever device design.^[^ ^297^ ^]^ Wang et al. coupled a humidity‐responsive, alkane‐inert GO layer with an alkane‐responsive, humidity‐inert PDMS layer for alternating actuation, which enabled the design of a shape‐changeable air valve with gas selectivity.^[^ ^298^ ^]^

PDMS is widely employed as the material for flexible substrates in various devices.^[^ ^299^ ^]^ For example, ultrasound patches formed by embedding a piezoelectric transducer within a conformable PDMS substrate can greatly enhance the transdermal absorption of small molecule drugs.^[^ ^300^ ^]^ The conventional application in this field is beyond the scope of this review and can be found elsewhere.^[^ ^16^ ^]^ Here we focus on one specific bimorph design: an electrically conductive layer on PDMS. Depending on the material of the conductive layer, the stretching of the PDMS substrate has an entirely different impact on the conductivity of the system, facilitating versatile applications.

**Figure 15:**
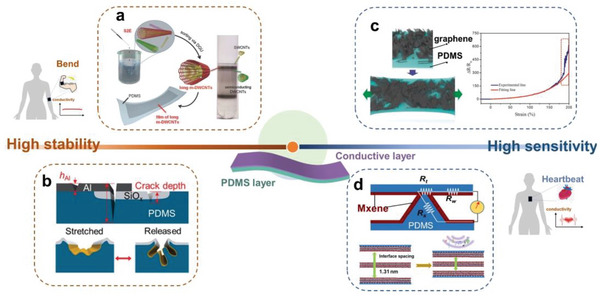
Flexible electronics based on a conductive layer on PDMS film. Highly stable conducting electrode under mechanical strain: a) metallic double‐walled CNTs to bridge the microscale gaps of the film formed in stretching process and maintain a high electrical conductivity, reproduced with permission.^[^ ^302^ ^]^ Copyright 2018, Wiley‐VCH; b) a junction‐free grid of expandable Au lines in the cracks which can be stretched in all directions while maintaining stable electrical conductivity. Reproduced with permission.^[^ ^304^ ^]^ Copyright 2021, Wiley‐VCH. Highly sensitive electrode for sensing: c) graphene with high defect density resulting in a rapid response of ΔR/R_0_; Reproduced with permission.^[^ ^305^ ^]^ Copyright 2018, Wiley‐VCH. d) Large layer spacing of MXene and the pyramidal array structure enabling dual amplification of the mechanical‐electrical signal for ultrasensitive sensing and real‐time voice classification. Reproduced with permission.^[^ ^306^ ^]^ Copyright 2020, American Association for the Advancement of Science.

Circuit boards consisting of stretchable electrical lines and substrates are considered pivotal components for wearable and implantable electronic devices,^[^ ^301^ ^]^ where the electrical properties are required to remain unchanged under significant external forces and extreme conditions. Wang et al. successfully prepared a stretchable and transparent conductive bimorph film using long, metallic, double‐walled CNTs as the conductive layer (**Figure** **15**a). The resulting bimorph film showed a high electrical conductivity of 1809 S cm^−1^ with 85% optical transmittance, which further increased to 3316 S cm^−1^ at 100% tensile strain and featured only a 4% decay after 1000 cycles. Scanning electron microscopy (SEM) analysis revealed that the long CNTs effectively bridged the microscale gaps formed in the film during stretching, maintaining intact electrical pathways throughout the film, thus preserving the overall conductivity.^[^ ^302^ ^]^ Rigid 1D fillers often suffer from uneven loading during the shrinkage of the substrate and may fracture prematurely. Kim et al. demonstrated that the modulus of polyvinylpyrrolidone (PVP)‐coated Ag NW networks could be manipulated via the swelling of PVP by various solvents (ethyl acetate, acetone, ethanol, water, etc.). Water is the most swelling solvent for PVP; thus, the modulus of PVP‐coated Ag NW chains decreased significantly by water swelling. The chains slid and rearranged into wavy structures more efficiently, enhancing the electromechanical stability of the film.^[^ ^303^ ^]^ Surprisingly, cracks on the substrate surface during stretching can be used as a template to prepare stable conductive paths as well. Kong et al. steam‐coated an Al film on the oxygen plasma‐treated PDMS surface, forming a SiO_x_ layer. As shown in Figure 15b, under stretching, cracks formed in the Al and SiO_x_ layer, and the deposition of Au grids on the cracked PDMS surface successfully produced a metal lattice circuit that could be stretched in all directions. The resulting thin film electrode exhibited a conductivity of 10^6^ S m^−1^ and negligible resistance change (Δ*R*/*R*_0_ ≤ 7%) at 125% biaxial strain.^[^ ^304^ ^]^

Highly sensitive electrical strain sensors play a pivotal role in smart wearable devices and structural services, and can be made from the identical bimorph architecture as discussed in the last paragraph, but with different filler designs. Pan et al. incorporated 3D graphene onto PDMS films for electrical strain sensing and found that low graphitization induces high sensitivity (Figure 15c).^[^ ^305^ ^]^ The microarray structure is capable of amplifying the mechanoelectrical sensitivity. An acoustic sensor was fabricated based on highly conductive MXene (Ti_3_C_2_T_x_) coated on a PDMS pyramidal array. The large interlayer spacing of MXene resulted in high mechanical sensitivity, and led to a dual enhancement when combined with a PDMS pyramid array (Figure 15d). The detected signal was 10 000 times higher than the noise level with a detection limit of 0.1 Pa. This pyramidal array‐based bimorph system showed higher accuracy for speech, with test data accuracy up to 95%, reaching the capability of commercial recorders.^[^ ^306^ ^]^ Similarly, Wan et al. sprayed a layer of CNTs on the surface of a PDMS microcone array for piezoresistive sensing and successfully incorporated the device into a bimodal artificial sensory neuron system.^[^ ^307^ ^]^

In addition to the above use of PDMS as a carrier for conductive materials, Kim et al. developed a method to control the two‐dimensional arrangement of Metal‐Organic Frameworks at a large scale (23 cm^2^) using thermally cured PDMS as the substrate. PDMS was mixed with casting solvent containing MOF particles and dispersed on the water surface. As the casting solvent evaporated, the PDMS concentration increased, and a thin sticky layer formed, keeping the aligned MOF particles in place. Eventually, the thermal‐cured PDMS layer produced a film with the fixed orientation of the MOF particles, which can be easily transferred to other substrates.^[^ ^308^ ^]^

## 4 Precision Fabrication of PDMS: New Chemistry and Cutting‐Edge Technology

While the compounding of PDMS with multifunctional fillers and the construction of their surface microstructure render PDMS additional functionality across a wide range of applications, the most commonly used PDMS is still thermal‐crosslinked Sylgard 184. Although the fabrication process is relatively simple, the required thermal curing^[^ ^309^ ^]^ and softness of the material hinder instant high‐resolution manufacturing. Therefore, there is a need to develop approaches that can rapidly produce PDMS 3D structures while ensuring that their properties are comparable or even better than the commercially available Sylgard 184.

### 4.1 Instant Curing

Besides the traditional thermal curing method, new chemistries coupled with other forms of external stimuli have been developed for rapid and feasible polymer crosslinking. A simple way to accelerate the curing process is to incorporate photothermal heaters as the filler material. Joseph et al. added Au nanoparticles in a PDMS prepolymer and achieved on‐demand crosslinking under illumination with a 10^9^‐fold increase in the curing rate.^[^ ^310^ ^]^

**Figure 16:**
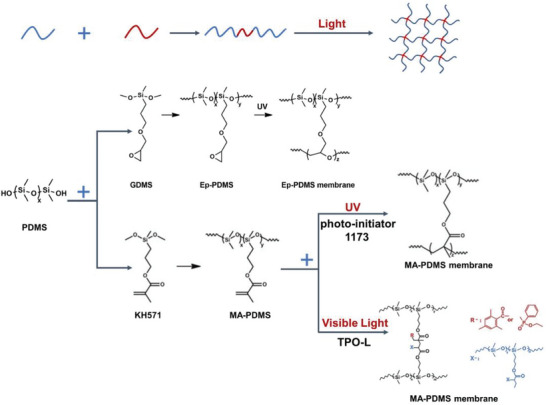
Developed crosslinking chemistry for photocurable PDMS. Blue curve represents a normal PDMS polymer chain, and red curve represents the polymer chain with a functional branch and acting as interconnecting sites after crosslinking. Molecular structures of PDMS, GDMS, Ep‐PDMS and Ep‐PDMS membrane: Reproduced with permission.^[^ ^312^ ^]^ Copyright 2020, Elsevier. Molecular structures of KH571 and MA‐PDMS. Reproduced with permission.^[^ ^311^ ^]^ Copyright 2019, Wiley. MA‐PDMS membrane and MA‐PDMS membrane: Reproduced with permission.^[^ ^313^ ^]^ Copyright 2022, Elsevier.

Photopolymerization of PDMS can be achieved by incorporating photoinitiators in PDMS prepolymers containing different functional groups (**Figure** **16**). Si et al. modified PDMS with 3‐methacryloxypropylmethyldimethoxysilane (KH571) to obtain methacrylate‐functionalized PDMS (MA‐PDMS), which could be polymerized in 30 s under UV irradiation after the addition of photoinitiator (2‐hydroxy‐2‐methylpropiophenone).^[^ ^311^ ^]^ However, free radical crosslinking is intrinsically susceptible to inhibition by oxygen. To circumvent this problem, the same research team introduced UV‐induced ring‐opening polymerization of epoxide groups into the PDMS crosslinking chemistry. PDMS was functionalized with epoxide (3‐glycidoxypropyl(dimethoxy)methylsilane), and the polymerization was completed in 200 s under UV light.^[^ ^312^ ^]^ Furthermore, PDMS can also cure under visible light with ethyl(2,4,6‐trimethylbenzoyl)phenylphosphonate (TPO‐L) as the photoinitiator, with a polymerization rate of 14 s^−1^.^[^ ^313^ ^]^

### 4.2 3D Printing

PDMS molding is a manual process that requires a relatively tedious assembly procedure. Since 2012, PDMS elastomers have been among the most investigated 3D printing materials,^[^ ^314^ ^]^ used in various techniques including direct ink writing (DIW),^[^ ^315^ ^]^ embedded 3D printing or freeform reversible embedding (FRE),^[^ ^314^, ^316^ ^]^ reduced photopolymerization or stereolithography (SL),^[^ ^317^ ^]^ and inkjet or material jetting.^[^ ^318^ ^]^ PDMS precursors are Newtonian fluids with shear rates in the range of 10^−1^ to 10^2^ s^−1^ and a constant viscosity of ≈0.9 Pa s;^[^ ^319^ ^]^ therefore, it is difficult to maintain the shape stability of printed PDMS. The PDMS ink state can generally be altered by sudden stress reduction, phase change, solvent evaporation, gelation, and polymerization of the ink. Once extruded, the viscoelastic fluid should rapidly recover or significantly increase its viscosity and modulus to maintain the printed architecture.^[^ ^320^ ^]^

In the DIW process, the ink is extruded from the nozzle and can be deposited into a specific pattern by the movement of the nozzle.^[^ ^321^ ^]^ Conventional wisdom holds the view that soft silicone such as PDMS is not suitable for 3D DIW as the printed ink pattern easily deforms by gravity, affecting the printing accuracy. Photocurable components such as PMMA can be added to PDMS prepolymer to form an ink that can be rapidly cured by UV light, which is essential for temporary gelation with a dense and stable state to finalize thermal crosslinking.^[^ ^322^ ^]^ In addition, FRE, an embedded 3D printing technique, has been explored for PDMS printing. In FRE, PDMS precursors were printed layer by layer in a support tank, and the polyacrylic microgel in the tank acted as a sacrificial hydrogel bath to provide physical support during PDMS curing, which can prevent the deformation of the ink.^[^ ^323^, ^324^ ^]^ Recently, Zhang et al. adopted electric‐field‐driven 3D printing to construct PDMS cylindrical microlens arrays. PDMS ink was deposited on the substrate in the form of narrow lines from the nozzle with high voltage and cured in a few tenths of a second while maintaining a stable structure.^[^ ^325^ ^]^

**Figure 17:**
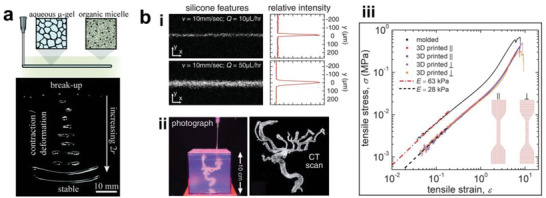
3D printing of PDMS in support materials. a) Elastic beams of microgels are 3D printed into viscoelastic fluid support baths. 3D printed beams with small feature sizes yield to smaller droplets or undergo deformation, while larger beams remain stable. Reproduced with permission.^[^ ^328^ ^]^ Copyright 2021, The Royal Society of Chemistry. b) Support material similar to PDMS is used in 3D printing. i) Linear characteristics of the printed PDMS; ii) Aneurysm model printed in the support material with AMULIT technology and scanned by computed tomography; iii) Tensile stress‐strain curves of printed specimens in different orientations showing linear stress–strain relationships at low strains. Reproduced with permission.^[^ ^329^ ^]^ Copyright 2023, American Association for the Advancement of Science.

The problem of collapsing and deforming of the printing structure can be solved by using embedded support materials. This material flows smoothly during printing, enveloping the deposited ink and ensuring print stability.^[^ ^326^ ^]^ A hydrophilic Carbopol gel, acting as a Bingham plastic, provides yield and fluidity, allowing the PDMS prepolymer to cure without deformation.^[^ ^323^, ^324^ ^]^ However, the interfacial tension between the ink and support material may lead to premature structure failure.^[^ ^327^ ^]^ Angelini et al. discovered that the stability of 3D printed beams within an elastic support material can be anticipated through the radius of the beam, the surface interfacial tension, and the yield stress of the solid. If the surface strain surpasses the yield stress, the fluid beam could break or deform (**Figure** **17**a).^[^ ^328^ ^]^ To address this, the same group developed the Ultra Low Interfacial Tension Additive Manufacturing (AMULIT), employing support materials chemically akin to PDMS inks. The resultant inverse emulsions hold water droplets suspended in a continuous silicone oil. The emulsion's rheological characteristics can be fine‐tuned, optimizing the filling fraction and droplet radius. Glycerol is added to achieve a refractive index match, facilitating macroscopic and microscopic imaging during printing. This ultra‐low interfacial tension offers the potential to print small structural features (8 µm in diameter, Figure 17b, i). Successfully crafted complex structures include cerebral aneurysms (Figure 17b, ii) and trilobular heart valves, exhibiting negligible modulus differences between variously oriented specimens (Figure 17b, iii). AMULIT can be used with a range of commercially available PDMS formulations to produce precise, durable devices, negating the need for specially formulated material inks.^[^ ^329^ ^]^

**Figure 18:**
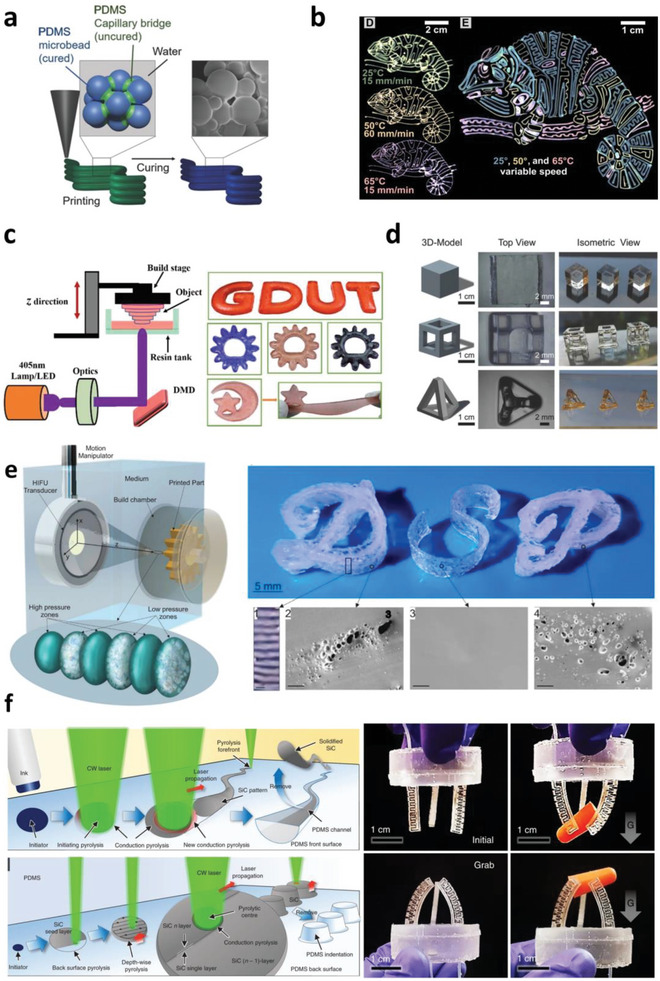
Additive manufacturing of PDMS by the state‐of‐the‐art 3D printing technologies, such as DIW (a, b), SL (c, d), DSP (e), and SLP (f). a) Capillary suspension ink possesses high storage moduli and yield stresses that are needed for DIW. Reproduced with permission.^[^ ^330^ ^]^ Copyright 2017, Wiley‐VCH. b) Introduction of a highly precise pneumatic extrusion system in DIW achieves adjustable structural color of PDMS‐b‐PLA copolymer solution. Sprayed PDMS‐b‐PLA shows different colors by self‐assembly after solvent evaporation. Reproduced with permission.^[^ ^331^ ^]^ Copyright 2020, American Association for the Advancement of Science. Both (c) and (d) are UV‐based SL of PDMS with different terminal groups: c) Mercaptoene and vinyl‐functionalized PDMSs gelated under UV illumination and then thermally cured. Reproduced with permission.^[^ ^133^ ^]^ Copyright 2018, Wiley‐VCH. d) Direct UV desktop‐SL 3D printing of PDMS‐S with similar property to that of Sylgard 184. Reproduced with permission.^[^ ^21^ ^]^ Copyright 2020, Elsevier BV. e) In the DSP process, a violent reaction occurs inside the bubble where the sound waves pass, leading to direct curing and deposition of PDMS. The resolution of the printing is regulated by the power of ultrasound. Reproduced with permission.^[^ ^333^ ^]^ Copyright 2022, Nature Publishing Group. f) Manufacturing 2D/3D PDMS through FSS or BSS. PDMS is partially converted to SiC by continuous wave laser irradiation from a light‐absorbing pyrolysis local initiation point. 2D or 3D PDMS were obtained after removing SiC. Reproduced with permission.^[^ ^9^ ^]^ Copyright 2021, Nature Publishing Group.

Usually, there exists a tradeoff between an ink's conformability (i.e., behaves like a solid material at low shear stress, storage modulus G′ > loss modulus G″) and its smoothness (i.e., has liquid‐like behavior at high shear stress, G″ > G′). However, it is possible to improve both properties by including functional additives. Roh et al. prepared a pure PDMS ink consisting of pre‐cured PDMS microbeads, PDMS prepolymer, and water as an external run‐off phase. As shown in **Figure** **18**a, the PDMS prepolymer binds to the microbeads by capillarity action to produce a thixotropic, extrudable gel‐like phase with high G′ and yield stress. After 3D printing and thermal curing, a stable 3D silicone structure was made, in which the pores created by the evaporation of water guaranteed elasticity and high flexibility.^[^ ^330^ ^]^ Polytetrafluoroethylene micronized powder^[^ ^319^ ^]^ or other particles can be used as the thixotropic agent to modulate the rheological properties of PDMS precursors to meet the requirements for 3D printing. Wang et al. prepared a phase change ink by mixing silica nanoparticles and wax particles (≈3 µm in average diameter) with PDMS. When the ink was extruded from a heated nozzle in a cold environment, the rapid disruption and reconstruction of the temperature‐sensitive wax phase (solid‐liquid‐solid) helped instantly control the rheological behavior of the ink. The G′ of the printed structure recovered to its initial value before melting, thus permitting shape‐holding at room temperature until PDMS polymerization occurred with a resolution close to 50 µm.^[^ ^320^ ^]^ Lai et al. synthesized a linear PDMS polymer backbone with abundant carboxylic acid groups (approximately one carboxylic acid group for every two O‐Si‐O‐groups), which can coordinate with Zn^2+^ to form Zn(II)‐carboxylate interactions. The polymer coordination equilibrium was very temperature sensitive, so the mechanical strength changed rapidly (within a few tens of seconds) and reversibly upon heating or cooling. It became a viscous liquid when heated to 120 °C and rapidly turned to solid when cooled, making it suitable for DIW.^[^ ^138^ ^]^

Interestingly, nonequilibrium self‐assembly can be combined with DIW to prepare bottlebrush block copolymer (BBCP) photonic crystals with tunable structural colors. Patel et al. prepared liquid inks by mixing PDMS‐block‐poly (lactic acid) (PDMS‐b‐PLA) BBCP with tetrahydrofuran (THF). The ink was sprayed onto the silicon substrate at different temperatures to form a thin layer by highly precise pneumatic extrusion. After the THF evaporated, PDMS‐b‐PLA self‐assembled into different structures and displayed diverse colors. By varying the temperature of the silicon substrate and the extrusion pressure (printing speed), the d‐spacing of BBCP can be varied with a maximum over 70 nm, corresponding to a reflection peak wavelength of 403–626 nm (blue to red) (Figure 18b).^[^ ^331^ ^]^

SL is an additive manufacturing technique that allows rapid shaping of photoinitiator‐doped PDMS into high‐resolution (≈50 µm) 3D structures.^[^ ^332^ ^]^ Liu's team prepared a 3D printable PDMS elastomer by the UV curing of mercapto‐alkene and vinyl‐functionalized PDMS, as well as thermal curing of other components in the ink (carboxyl‐alkene and amide‐functionalized PDMS). The elastomer itself possessed inherent viscoelasticity (G″ > G′), while the gelation observed as a result of G′ > G″ realized in 3 s under UV light precisely met the prerequisites for 3D SL printing (Figure 18c).^[^ ^133^ ^]^ Bhattacharjee et al. demonstrated desktop‐SL 3D printing of PDMS resin under 385 nm UV light by adding a photoinitiator (TPO‐L) to telomeric methacryloxypropyl‐PDMS (abbreviated as PDMS‐E) or poly (methacryloxypropyl siloxane‐co‐dimethylsiloxane) (abbreviated as PDMS‐S) and utilizing a UV‐blocker (e.g., isopropyl‐thioxanthanone) to ensure a satisfactory *z*‐resolution of 12.5 µm. The authors adjusted the molar ratio between PDMS‐E and PDMS‐S and printed PDMS with a maximum Young's modulus of 937 kPa or maximum elongation at a break of 153% (Figure 18d).^[^ ^21^ ^]^

Selective laser sintering (SLS) is a powder‐based additive manufacturing technique that uses a laser to fuse the deposited powder to form printed parts based on 3D model data. Powder‐based 3D printing of PDMS remains a significant challenge due to the inherent thermosetting nature of PDMS. Sun et al. obtained a covalently adapted network (CAN) by including dynamic pyrazole urea bonds. Kilo‐scale PDMS powders were prepared for SLS by cryogenic grinding, and PDMS CANs became thermoplastics through dynamic covalent bond dissociation or exchange reactions under thermal spurs. The printed PDMS CANs containing 3‐amino‐5‐tert‐butylpyrazole exhibited Young's modulus of up to 21 MPa.^[^ ^120^ ^]^

Recently, Habibi et al. successfully printed pure Sylgard 184 PDMS, using an approach called direct sound printing (DSP). Complex geometries with zero to variable porosity and 280 µm feature size can be printed. As shown in Figure 18e, a chemically active acoustic cavitation region was created where the ultrasonic wave passed by PDMS, causing an extraordinarily intense and short‐lived (in picoseconds) reaction at the interior of the bubble, with a temperature spike to ≈15000 K and a pressure more than 1,000 bar, under which the PDMS was instantly cured and deposited onto the platform. Ultrasound as well as other parameters of the building material affect the microstructure of the printed parts. For example, ultrasound at 20 W power can print fully transparent PDMS, while porous PDMS printed at 40 W is opaque.^[^ ^333^ ^]^

In contrast to all previous forms of 3D printing, successive laser pyrolysis (SLP) was used to develop monolithic quasi‐3D digital patterning of PDMS. SLP includes front surface scanning (FSS) and back surface scanning (BSS), both of which start from a light‐absorbing pyrolysis local initiation point and partially convert PDMS to SiC by laser irradiation. 2D or 3D PDMS were obtained after removing SiC (Figure 17f). FSS was able to generate a surface roughness reaching 1 nm on PDMS. However, the originally generated SiC prevented the laser from penetrating deeper, bringing fundamental limits to the high aspect ratio or deep patterning. In contrast, BSS created a pyrolysis initiation point on the backside of PDMS and built a SiC layer by layer inside PDMS, which enabled pyrolysis molding within PDMS. The combination of FSS and BSS can transform a bulk elastomer directly into tangible devices in a short amount of time (1 h).^[^ ^9^ ^]^

In most applications, PDMS is used in the form of a bulk material or thin film, while much less attention has been paid to PDMS fibers. Fibrous PDMS is expected to possess a large aspect ratio, high surface area, high porosity, and good permeability. It is difficult for a Sylgard 184‐like PDMS to conform with fiber spinning standards, which hampers the production of stable and high‐performance fibers. However, there have been encouraging advances in recent years. The tube mold method successfully prepared short PDMS fibers with micrometer lengths.^[^ ^334^ ^]^ Typically, PDMS precursors were injected into the tube mold, and the thermally cured PDMS was separated from the mold afterward.^[^ ^335^, ^336^ ^]^ Shang et al. injected a mixture of PDMS and ethylene glycol droplets containing carbon‐encapsulated Fe_3_O_4_ nanoparticles into a polytetrafluoroethylene tube and exfoliated it after curing to obtain magnetic responsive fibers.^[^ ^337^ ^]^

Compared with the template method, the spinning methods (wet spinning and electrospinning) provide enormous advantages and appealing opportunities. Jeong et al. successfully prepared long PDMS fibers (in meters) with a consistent width by a thermally induced wet spinning method. PDMS solution extruded from a syringe crosslinked in an oil coagulation bath between 180 and 230 °C and stretched at a speed of 1.2–12.5 m min^−1^. This solves the problems associated with the generation of solvent vapors when using conventional wet spinning.^[^ ^338^ ^]^ Electrospinning enables the preparation of PDMS fibers at relatively low temperatures. PMMA can be added to the PDMS precursor to enhance the chain entanglement for better electrospinning.^[^ ^339^ ^]^ Furthermore, PDMS fiber with a core‐shell structure was electrospun on a hot (100 °C) plate electrode, and the PVP shell was removed afterward by ethanol. PDMS fiber mats, benefiting from the structural flexibility, can be stretched to 403% of their initial length, with a significantly larger elongation at break than individual PDMS fibers.^[^ ^340^ ^]^

## 5 Conclusions and Outlook

Coinciding with the rapid development of soft materials and flexible electronics, PDMS—the simplest and most common silicone compound—has transformed from the central component in children's toy—silly putty, to the material of choice for many emerging technologies such as flexible electronics, bioengineering, soft actuators, implantable sensors, soft robotics, and superwetting surfaces. This review systematically examines the latest progress through three explosively developed areas, i.e., the bulk composites, surface modification, and 3D printing of PDMS‐based functional elastomers. Substantial and encouraging progress has been made, especially within the last several years. Integrating the latest advances in nanotechnology and soft materials has boosted the development of PDMS‐based functional elastomers, with completely tunable physical attributes and tailored chemical properties. The fundamental principles for fabricating PDMS functional composites have been established through various approaches to control the incorporation of fillers that impart specific functionalities and stimuli‐responsive capabilities. The introduction of magnetic and conductive fillers equips PDMS with novel properties that otherwise do not exist in the virgin material. Additionally, the high optical transparency and ultralow fluorescence of PDMS enable the fillers to fully define the optical performance of the composite material. Another characteristic achievement is the design of self‐healing PDMS composites, which with slight physical and chemical modifications, display “life‐like” programmability. Surface functionalization and multiscale micro‐ and nanostructures can introduce other novel functionalities and wetting capabilities, such as superhydrophobic, hydrophilic, self‐cleaning, de‐icing, and adhesion properties, which in some cases can mimic the behavior of biological interfaces. The compatibility, high stretchability, and high CTE make PDMS film uniquely advantageous for bimorph soft actuators. Furthermore, besides the traditional thermal crosslinking of vinyl‐terminated PDMS, photo‐crosslinking and 3D printing of PDMS allow instant production and high‐resolution manufacturing, opening new avenues toward a broader range of applications.

Driven by an in‐depth understanding of fundamental physics, chemistry, and materials science, the development of the PDMS functional elastomer has precipitated many exciting breakthroughs. For example, there usually exists a tradeoff between stiffness and extensibility for traditional, covalently crosslinked polymers. However, the recent progress in materials science, compounding methods, and functionalization chemistry have also made it possible to successfully overcome this challenge and produce PDMS‐based elastomers with superior properties in both stiffness and extensibility.^[^ ^103^, ^104^, ^105^ ^]^ Another example is the biomimic functional microstructured surfaces. Scientists not only can emulate the plants and animals (e.g., lotus leaf, gecko, tree frog, octopus, etc.) in nature, but they have also developed devices and systems that surpass these natural counterparts, as evidenced by exciting innovations in PDMS‐based smart interfaces and devices which are well‐adapted to diverse environments.

Though remarkable progress has been achieved, more fundamental research is needed to address existing challenges and explore new frontiers. Many questions are still open. For example, researchers found that some functional fillers, such as organic molecules, are often incompatible with the conventional hydrosilylation crosslinking reaction for vinyl‐terminated PDMS.^[^ ^22^ ^]^ Therefore, preparing a PDMS film with a specific color and high level of transparency is more challenging than expected. However, with the recent advances in nanomaterials processing^[^ ^341^ ^]^ and nanocomposite chemistry, one can expect exciting new functions to emerge from a well‐bonded marriage between PDMS and the many available functional nanomaterials.

Intensive interest in PDMS surfaces customized with micro‐ and nanostructure arrays has arisen because these materials hold great promise to provide a versatile micro/nano platform for surface mass transport, chemical reactions, lithography, and signal collection. The unique advantages associated with these arrayed surfaces include: i) a contact surface with a substantial specific area and tunable physical and chemical properties; ii) a small shape factor with an ultrahigh sensitivity to deformation; iii) parallel addressability with high resolution; and iv) a specific structural response to magnetism, light, and other forms of electromagnetic waves. The platform can control (increase/suppress) three‐phase contact regions and microfluidic dynamics, optimize the reactants' and products' mass transport kinetics, and facilitate interfacial chemical reactions. In addition, the platform enables various types of actuating and sensing capabilities via the integration of diverse stimuli‐responsive fillers or coatings. Typical examples are flexible pressure sensors, but the potential is well beyond them and remains largely unexplored. Furthermore, inspired by the efforts toward multiplexed, array‐based techniques in cantilever‐free scanning probe lithography allowing the writing of diverse features between neighboring pens,^[^ ^22^, ^96^, ^342^ ^]^ the ability to direct the action of individual microstructures in an array opens up exciting new prospects, enabling site‐specific administration, high‐throughput sensing, and material manipulation. Thus, it is possible to transform the functional surface array into a general micro‐/nanoscale platform with high production abundance and low costs. This highly sought‐after goal has vast potential for exploration in the future.

Additive manufacturing of PDMS‐based functional materials and devices has jumped into the limelight due to the attractive application potential in areas including, but not limited to, soft actuators, microrobots, flexible electronics, and bioengineering. Undoubtedly, it is highly rewarding to keep refueling and propelling the development of this field, and the efforts will eventually benefit practical industrial production. Considerable efforts have been devoted to the rheological tuning of PDMS composites to make PDMS amenable to various 3D printing criteria. The exploration of instant curing is also ongoing. Rapid crosslinking has still been largely limited to UV‐assisted strategies, which threaten the environment and human health. The ability to fabricate an optically crosslinked PDMS elastomer as easily as Sylgard 184 will unlock a host of remarkable possibilities in manufacturing and applications. We expect more breakthroughs in these areas in the future. Furthermore, incorporating PDMS in spinnable or wearable materials will also pave the way for functional fibers and smart textiles. In general, PDMS is a brilliant research and development (R&D) material for various communities, however, translating R&D efforts into large‐scale manufacturing for consumer application is quite challenging. It makes sense that thermally processable elastomers (e.g., thermoplastic polyurethane, TPU) are more in line with standards for scalable manufacturing. Nevertheless, the exciting breakthrough in precision fabrication of PDMS provides vast opportunities in multidisciplinary applications.

While these challenges continue to exist and others are sure to emerge, the field of functional PDMS elastomers is well‐positioned to facilitate new sciences and be utilized in innovative new designs, devices, and applications. We believe that functional PDMS elastomers possess tremendous potential in chemistry, physics, biology, materials science and engineering, and information technology.

## Conflict of Interest

The authors declare no conflict of interest.
